# Functional Bio-Based Polymeric Hydrogels for Wastewater Treatment: From Remediation to Sensing Applications

**DOI:** 10.3390/gels10080498

**Published:** 2024-07-27

**Authors:** Giulia Rando, Elisabetta Scalone, Silvia Sfameni, Maria Rosaria Plutino

**Affiliations:** 1 Institute for the Study of Nanostructured Materials, ISMN—CNR, URT of Messina, c/o Department of ChiBioFarAm, University of Messina, 98166 Messina, Italy; giulia.rando@cnr.it (G.R.); elisabetta.scalone@studenti.unime.it (E.S.); 2 Department of Chemical, Biological, Pharmaceutical and Environmental Sciences (ChiBioFarAm), University of Messina, 98166 Messina, Italy

**Keywords:** bio-based hydrogel, functional material, wastewater treatment, sensing, environmental remediation

## Abstract

In recent years, many researchers have focused on designing hydrogels with specific functional groups that exhibit high affinity for various contaminants, such as heavy metals, organic pollutants, pathogens, or nutrients, or environmental parameters. Novel approaches, including cross-linking strategies and the use of nanomaterials, have been employed to enhance the structural integrity and performance of the desired hydrogels. The evolution of these hydrogels is further highlighted, with an emphasis on fine-tuning features, including water absorption capacity, environmental pollutant/factor sensing and selectivity, and recyclability. Furthermore, this review investigates the emerging topic of stimuli-responsive smart hydrogels, underscoring their potential in both sorption and detection of water pollutants. By critically assessing a wide range of studies, this review not only synthesizes existing knowledge, but also identifies advantages and limitations, and describes future research directions in the field of chemically engineered hydrogels for water purification and monitoring with a low environmental impact as an important resource for chemists and multidisciplinary researchers, leading to improvements in sustainable water management technology.

## 1. Introduction

With growing environmental pollution and the scarcity of available water resources, the treatment of industrial and citizen wastewater has been the focus of several global policies and scientific investigations [1]. Currently, different industrial wastewater resource treatment approaches are employed, such as ammonia desulfurization, ion exchange membranes, chemical precipitation, etc., but these technologies have the disadvantage of employing complex and time-demanding processes, or giving rise to secondary pollution phenomena, to mention some [2,3]. 

In this regard, wastewater treatment has several issues when dealing with the presence of emerging pollutants (i.e., pharmaceuticals, organic dyes, fertilizers, herbicides, etc.) that are not yet normally handled by standard wastewater treatment techniques. In particular, some of the major challenges associated with emerging contaminants in wastewater treatment include: (i) a limited regulatory framework; (ii) analytical detection and monitoring; (iii) treatment technologies; (iv) transformation products; (v) mixtures and synergistic effects between contaminants; (vi) resilience to climate change; (vii) a lack of public awareness; and (viii) urbanization and population growth [4,5,6]. 

New innovative functional materials are constantly developed to improve the efficiency of industrial wastewater treatment, solve the water resource crisis, and realize coordinated ecological development [7]. Moreover, addressing these difficulties needs a multifaceted strategy that includes study, regulatory action, technology innovation, and public awareness. Collaboration among scientists, policymakers, water utilities, and the public is also required to find long-term solutions for managing emerging pollutants in wastewater. In these aspects, filtration approaches (i.e., membrane-based and activated carbon-based) are frequently utilized techniques for wastewater cleanup, but they still have certain limits and research gaps that must be understood and identified in order to advance in the application sector. Some examples include: (i) fouling; (ii) selectivity and flux trade-off, (iii) chemical and biological stability, (iv) energy consumption, (v) costs and scaling challenges, (vi) material sustainability, (vii) emerging contaminants treatment, and (viii) life cycle assessment and cost-benefit analysis [8,9,10,11].

Furthermore, advanced material optimization necessitates a thorough understanding of contaminant properties, a focus on selectivity and durability, and the creation of low-cost solutions. This general approach is critical for developing filtration technology and making considerable progress in the removal of new contaminants from water sources, as well as “common” pollutants. Closing these research gaps would not only increase the efficiency and effectiveness of filtration technologies for wastewater remediation, but it could also pave the way for the development of more sustainable, less expensive, and innovative solutions for more inclusive and efficient water treatment.

In this context, molecular gels have shown great potential in water treatment due to their large surface area, which allows for efficient absorption of pollutants, and their ease of customization and production. Several research studies have demonstrated the effectiveness of these polymeric gel-based materials in removing a range of contaminants, including oil, heavy metals, dyes, and medicinal compounds [12,13,14]. 

Several key factors of molecular gels need to be considered viable for their practical use, including their physical properties and ability to be recycled, in order to overcome the hurdles that stand in the way of their implementation in real-world applications [15]. 

As a class of polymeric material, hydrogels are three-dimensional (3D) cross-linked polymer chains that are able to absorb and trap a significant amount of water by expanding in an aqueous environment without dissolving. Recently, the development of hydrogels has attracted the interest of researchers from different research and industrial fields, with the purpose of solving numerous challenges using hydrogel swelling capacity [16]. As a matter of fact, the ability of hydrogels to absorb water derives from hydrophilic functional groups such as hydroxyl, carboxylic acid, imide, sulphonic acid, and amide present in the polymeric main chain, while cross-links connecting the polymer structure prevent hydrogel from dissolution. These polymeric structures can contain various amounts of water depending on the nature, qualities of the polymer employed, and density of the network [17].

Hydrogels are mainly classified based on a variety of factors, including their starting source and cross-linkers [18]. In terms of their source, hydrogels can be natural (or bio-based) or synthetic. 

Chitosan, collagen, alginate, agarose, hyaluronic acid, gelatine, fibrin, etc. are some examples of natural polymers able to form hydrogels presenting intrinsic biocompatibility and biodegradability, but unfortunately still low stability and mechanical resistance; otherwise, synthetic hydrogels, such as poly vinyl alcohol (PVA), polyacrylic acid (PAA), etc., have a long service life and a high capacity for water absorption [19,20]. However, recent approaches rely on the design of hybrid and composite hydrogels (either incorporating both natural and synthetic components and/or organic and inorganic polymers) in order to achieve better and improved performances [21].

According to the cross-linking mechanism, hydrogels can be classified into physical and chemical hydrogels. (i) Physical cross-linked hydrogels, known as reversible gels, have gained importance due to their relative ease of production and the advantage of not using cross-linking agents during the synthesis (physical reversible cross-linking can occur via hydrogen bonding, stereo complex formation, ionic interaction, or using repetitive freeze-thaw cycles). (ii) Chemical cross-linked hydrogels are obtained by a synthetic approach including reactions between complementary functional groups (Schiff base formation, Michael addition, condensation, etc.), or by ultraviolet light, through free-radical polymerization and high-energy irradiation, and it is a preferred method since it employs covalent bonds between functional groups into polymer chains [22]. Actually, a cross-linking mechanism is important to prevent the crushing of the hydrogel during the swelling process, giving rise to final materials with greater physical integrity and mechanical strength. Compared to physically cross-linked hydrogels, chemically cross-linked ones feature a more stable network with relatively high mechanical strength and long degradation times; however, cross-linking agents are often toxic and not environmentally friendly compounds [18].

In the last few decades, hydrogels have received a lot of interest thanks to their unique properties, which make them useful in a wide range of applications such as the food industry, drug delivery, regenerative medicine, and environmental remediation. Nutritional quality and food protection are extremely critical in the food industry. For example, there is an increasing search for biodegradable substitutes for synthetic materials in order to improve the stability, safety, and quality of packaged foods. In this regard, it has also been found that cellulose-based hydrogels may be used to monitor food quality as freshness indicators that detect pH changes, chemical degradation, and microbiological development based on metabolite synthesis in the food [23].

Moreover, since their early development in scientific research, hydrogels have been recognized as suitable for contact either with the skin or the human body due to their similarity with the extracellular matrix. In fact, in the field of regenerative medicine, injectable hydrogels have emerged as promising biomaterials; they are used to replace lost or damaged natural tissue due to their ease of manipulation, complete filling of surgical areas, and good permeability [24,25].

Nowadays, hydrogels are also of great interest because they are employed as an excellent system for the delivery of different types of drugs to the target sites; indeed, thanks to their interesting physical properties, in particular biocompatibility and porosity, they are able to encapsulate, protect drugs, and provide sustained and targeted release, at the same time increasing the effect of the drug itself and reducing side effects [26]. 

Recently, hydrogels have been identified as one of the most promising solutions for next-generation solar evaporation technology for generating freshwater from non-potable water and desalinating against pathogens [27]. As discussed by Parsa et al. [28], solar-based water desalination systems are being researched as a cost-effective and eco-friendly method for providing clean drinking water. The use of nanostructured materials and hydrogels in solar-powered stills and interfacial evaporation systems is thought to be a highly effective way of enhancing their efficiency. 

Bio-based hydrogels have also emerged as a competitive alternative for addressing the urgent worldwide challenge of water pollution, both in terms of sensing and contaminant removal in water treatment or remediation sectors. Furthermore, the implications of functional and/or stimuli-responsive bio-based hydrogels lie in their unique properties and capabilities, which make them highly versatile materials for a wide range of applications in various fields [29,30]. 

Ongoing research in this area aims to push the boundaries of functional hydrogel development, leading to advanced and innovative materials and technologies with improved performance and sustainability [31]. As research and industrial activity efforts continue, the impact of these innovative bio-based materials is expected to grow, contributing to advancements in healthcare, environmental sustainability, and high-tech textiles and cosmetics, driving innovation and technological advancement in materials sciences, polymer chemistry, and materials engineering.

This review aims to show the design, synthesis, and applications of functional bio-based hydrogels as a promising avenue for emerging wastewater treatments, addressing the actual pressing global challenge of water contamination. 

In this context, the next paragraphs will discuss the application of bio-based hydrogels in (waste)water remediation and sensing, including their advantages and disadvantages and a comparison of the different sorption/detection mechanisms for various water contaminants and environmental parameters. Moreover, the development of hybrid/composite hydrogels based on different nanomaterials is explored, highlighting the features and characteristics of the different classes of compounds employed as dopant agents. 

The interdisciplinary nature of this review, combining materials science, chemistry, and environmental engineering, may provide a concise overview of recent advances in creating tailored functional hydrogel systems that may open the way significantly to high-performing water purification and sensing technologies. In particular, this review shows the key role of bio-based functional hydrogels in their ability to address complex challenges across various environmental sectors while offering sustainable, customizable, and smart solutions for wastewater treatment.

## 2. Bio-Based Polymeric Hydrogels for (Waste) Water Treatment

### 2.1. Bio-Based Hydrogels for Environmental Remediation

Hydrogels for water remediation present different advantages due to their easy regeneration, allowing for the reuse and recovery of adsorbed pollutants. Recovering polluting resources, such as precious metals and dyes, is not only economically and ecologically beneficial but also allows for recycling in industrial and manufacturing processes. Currently, there is limited research on hydrogel regeneration beyond five adsorption-regeneration cycles in laboratories. Further effort in hydrogel research prioritizes optimum regeneration time, material stability, and regeneration conditions (e.g., temperature, solution nature, and concentration). In this regard, the regeneration solution, often composed of mineral acids and bases, should be inexpensive, plentiful, simple to create, and non-hazardous, depending on the nature of the adsorbed pollutant and the hydrogel chemical composition [15].

Moreover, the development of eco-friendly hydrogels for water remediation starting from non-fossil-based compounds, biopolymers, natural derivatives, secondary raw materials, or water-based formulations is currently explored by researchers [32,33,34]. 

Traditional biopolymer hydrogels are, for example, based on starch, cellulose, chitosan, alginate, gums, dextran, pectin, carrageenan, etc., which allow the production of hydrophilic materials with a three-dimensional network and porosity [35,36,37].

Aromatic-free and low-impact, environmentally friendly supramolecular hydrogels that efficiently entrap aqueous contaminants can be obtained as well, starting from peptides. In particular, Giuri et al. developed gels by combining the aromatic-free tripeptide Boc-L-Ala-Aib-L-Val-OH in water and alcohol solutions. The hydrogel produced in 2-propanol/water may absorb up to 97.0% of Eosin Y and 88.6% of diclofenac sodium from aqueous solutions. Moreover, the obtained hydrogel is thixotropic, thermoreversible, and biocompatible [38].

The most relevant systems that will be described thereafter are summarized in Table 1, in which the starting materials and performances of the developed hydrogels are compared.

#### 2.1.1. Nanomaterial-Doped Bio-Based Hydrogels for Pollutant Sorption

Considering the current wastewater treatment issues and limitations, continuous advancements in the field of nanotechnology and material sciences represent the necessary skills to maintain a cutting edge in the design and development of environmentally friendly nanostructured materials and smart technologies for water filtration, bearing enhanced functionalities, specific porosity, selectivity towards water contaminants, and recyclability. Nanoparticles featuring extraordinary physical and chemical properties (i.e., increased reactivity, high surface-to-volume ratio, and unique surface chemistry) have led to innovative solutions to a variety of environmental concerns [53,54,55,56,57,58,59]. Specifically, the advancement of research in this field involves the creation of novel nanocomposites and hybrids with different functional characteristics and the ability to be easily regenerated and reused. This represents significant progress due to the combination of nanomaterial properties with environmentally friendly methods for addressing environmental issues and the circular economy, thereby avoiding the generation of waste or secondary pollution [57,60,61,62,63].

With this in mind, the rational design of filtering/adsorbing functional materials often resulted in the implementation of synthetic procedures based on selected (blended) polymeric matrices, such as natural polymers or synthetically polymerized engineered (organic, inorganic, hybrid, and composite) materials. The combination of hydrogel and nanoparticles may synergistically increase the performance of the resultant hybrid/nanocomposite materials and improve their physical, chemical, and biological properties, that is, displaying increased strength and flexibility [64,65]. 

In these regards, various types of nanoparticles (i.e., inorganic, carbon-based, silica-based, and polymeric) have been incorporated into numerous hydrogels derived from natural polymers [66,67]. Furthermore, the nature of the incorporated nanoparticles determines how the final functional hydrogels may be used for environmental remediation, leading to systems useful for chemical sensing, adsorbents, and photocatalyst materials [68]. 

Nanoparticle size, shape, chemical composition, and functionalization may all be easily adjusted to increase the functional hydrogel selectivity and efficiency. The eco-friendly nanocomposite hydrogels have the potential to act as scavengers for heavy metals and synthetic organic molecules, such as dyes, halogenated compounds, herbicides, and insecticides, or as soil conditioners and ion exchangers [21].

**Figure 1:**
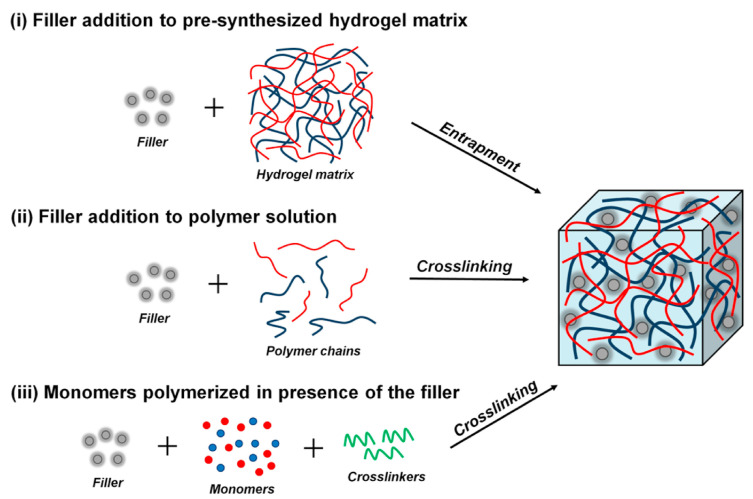
An illustrative system summarizing the main methodologies utilized to create composite hydrogels including the entrapment of the fillers in a pre-formed hydrogel matrix (i), combining fillers with cross-linked polymer solutions (ii), the simultaneous polymerization and cross-linking of monomers with fillers (iii). Reprinted with permission from Ref. [69]. Copyright 2021, Antonio G.B. Pereira, Journal of Cleaner Production, Elsevier.

There are different approaches for hybrid/composite hydrogel development doped with proper nano- and micro-structured fillers, as summarized in Figure 1. 

In particular, one approach (i) in Figure 1 could be the entrapment of the fillers in a pre-formed hydrogel matrix by their sorption into the matrix. This technique has been thoroughly evaluated to synthesize metallic nanoparticles in situ using the hydrogel matrix to decrease metallic ions and stabilize the nanoparticles. Another approach (ii) in Figure 1 involves combining fillers with cross-linked polymer solutions. In this situation, blended polymers may be physically or chemically cross-linked to the filler. The third technique (iii) in Figure 1 involves the simultaneous polymerization and cross-linking of monomers (mostly acrylic monomers) in the presence of reactive or unreactive fillers [69].

By combining MNPs with polymeric matrices, innovative nanocomposites or nanohybrids with fascinating and practical attributes can be obtained. Metal nanoparticles (MNPs) are nanomaterials with unique physical and chemical properties compared to larger bulk materials, attributed to their small size and high surface-to-volume ratio. These MNPs exhibit antibacterial properties as well as significant optical, electrical, and catalytic characteristics. In this context, they enable the creation of diverse nanocomposites, including polymer–matrix composites wherein individual nanoparticles are finely distributed, composite nanoparticles such as core/shell or surface-modified nanoparticles, and microsphere composite nanoparticles that are larger composite spheres [70,71,72]. 

Among their different applications, recent studies involve the use of different types of metal nanoparticles (i.e., gold, silver, and titanium dioxide) in black paint (nano-paint) production for solar desalination absorbers and the obtaining of clean water. Using solar panels in water desalination is a popular strategy for generating electricity and improving system performance. However, when the cost of systems and the finished output rise, passive approaches become a more attractive alternative to active systems. In this regard, metal nanoparticle-based black paint with the optimal fraction of nanoparticles coated at the interface of solar desalination absorbers was revealed to be critical to overcoming this limit, as studied by Parsa et al. [73].

Metal nanoparticles are also efficiently employed in emerging contaminant removal from water through sorption processes [57]. A hydrogel nanocomposite (HNC) based on sustainable karaya gum cross-link poly(acrylamide-co-acrylonitrile) and embedding silver nanoparticles was efficiently developed by Pandey et al. and tested for the removal of synthetic dye from industrial water using crystal violet model contaminants. The major goal of incorporating AgNPs into the hydrogel matrix consisted of improving of the hydrogel’s structural’ and mechanical characteristics, as well as its adsorption capacity. The obtained hydrogel revealed a high adsorption capacity of a 50 ppm crystal violet solution after a contact time of 360 min (Q_max_ = 1000 mg·g^−1^, sorbent dosage = 0.02 g, T = 298 K, and pH = 8) and recycling capability [39].

The different size, shape, and morphology of metal-based nanoparticles can influence the properties of the obtained hydrogels. A comparison of nugreek galactomannan-based green hydrogels (modified with 2-octenyl succinic anhydride) doped with Ag nanoparticle clusters with a core–shell or rod-shaped structure is given by Liu et al. In particular, Klason lignin was sulfonated to obtain sodium lignosulfonate and used to synthesize core–shell lignin-Ag NPs named KL-Na-Ag NPs.

The same procedure was employed to obtain rod-shaped lignin-Ag NPs (OL-Na-Ag NPs) starting from sulfonated acetosolv lignin. KL-Na-Ag NPs and OL-Na-Ag NPs were finally utilized as initiators to obtain bio-based hydrogels by free-radical polymerization. In this regard, the core–shell KL-Na-Ag NPs (particle size 15.5 nm) have shown capability in creating bio-based hydrogels by producing free radicals and attacking the double bond structure of octenyl succinic anhydride. Moreover, lignin-Ag NPs may establish a dynamic catechol redox mechanism in the hydrogel internal network, allowing catechol groups to be generated continually. These functionalities give the hydrogel self-healing, electrical conductivity, and antimicrobial properties. In fact, the hydrogel demonstrated antibacterial action against *S. aureus* and *E. coli*. Therefore, these hydrogels may potentially be suitable materials for smart flexible systems as well as for water remediation purposes [74].

Metal-organic frameworks (MOFs) have received a lot of attention over the years as traditional organized porous solids. MOFs are porous materials based on metal ions or oligonuclear metallic complexes bonded together with organic ligands. Among the great porosities, MOFs possess a large specific surface area and good thermal stability. 

Moreover, the pores may host neutral molecules (i.e., solvent molecules, ions, gas molecules, and biomolecules) [75]. As chemical synthesis progressed and became more adaptable, several applications of these functionalized materials were expanded in various fields due to their chemical adaptability, nanoscale with tailored cores, and surfaces. Iron-containing MOFs, among many other MOFs used for environmental remediation, have sparked widespread attention due to a combination of semiconductor characteristics and the Fenton process [76]. Combining different metal nanomaterial effects, Duan et al. created a flexible bio-based hybrid hydrogel out of Ag NPs@ MIL-100(Fe) photocatalysts and guar gum (GG) by simple mixing and self-crosslinking. 

**Figure 2:**
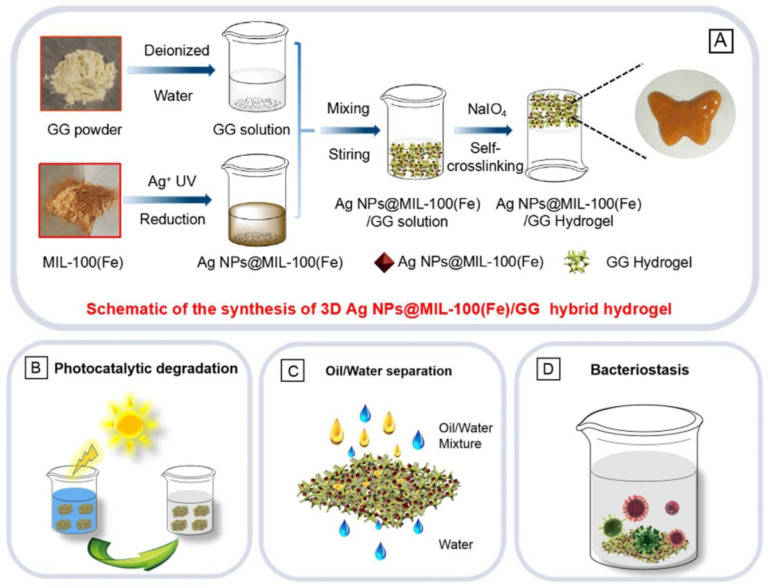
Synthesis (**A**) and applications of the multifunctional 3D Ag NPs@MIL100(Fe)/GG hybrid hydrogel for photocatalytic degradation (**B**), oil/water separation (**C**) and bacteriostasis (**D**). Reprinted with permission from Ref. [40]. Copyright 2020, Chao Duan, Carbohydrate Polymers, Elsevier.

The hybrid hydrogel incorporates multifunctional dye adsorption and degradation, oil/water separation, and antimicrobial characteristics, allowing for use in complicated wastewater treatment processes (Figure 2). The inclusion of Ag nanoparticles (Ag NPs) not only increased the photocatalytic activity of MIL-100(Fe) but also provided antibacterial activity to Ag NPs@MIL-100(Fe)/GG hydrogel. 

In particular, the bio-based GG hydrogel scaffold demonstrated excellent injectability, remodeling, and self-healing properties, allowing for effective oil/water separation (silicone oil, cyclohexane, and canola oil) and facilitating the nano-photocatalyst powders recyclability/sustainability. Adding Ag NPs significantly improved the photocatalytic activity of MIL-100(Fe), resulting in nearly 100% removal of the organic dye after 100 min of irradiation (MB concentration: 40 mg/L and 40 mL). Moreover, the as-prepared hydrogel demonstrated an efficient oil/water separation capacity, with separation efficiencies of 99.10% for silicone oil, 97.82% for cyclohexane, and 98.61% for canola oil [40].

On the other hand, MOFs were revealed to not be very performant sorbents for phosphate removal or recovery (with high concentrations of phosphate) because of their high linker release, low reusability, and possibility of secondary linker-related contamination [77]. In this regard, layered double hydroxide (LDH) has seen widespread use in anion pollution remediation due to its high porosity, surface area, and ion exchange capacity. Moreover, because of their unique physical and chemical qualities, rare earth compounds have demonstrated a very good ability to remove phosphate from water. In particular, lanthanum (La) and cerium (Ce) are frequently used because of their strong affinity and selectivity for phosphate [78,79]. 

For example, Quing et al. developed a rare earth-based LDH-embedded chitosan hydrogel bead for the removal of phosphate from water. In particular, two types of rare earth-based layered double hydroxide/chitosan (CS) hydrogel beads were developed employing La and Ce rare earths (namely, LaCa-LDH/CS and CeCa-LDH/CS, respectively). The combination of CS and LDH revealed that it was critical to achieve better adsorption capacity than individual LDH, owing to the incorporation of -NH_2_ functionalities and the uniform distribution of LDH in CS. LaCa-LDH/CS and CeCa-LDH/CS sorption results demonstrated maximal phosphate adsorption capacities of 149.5 and 174.6 mg P/g, respectively, employing 200 mg P/L and pH 5, performing better than pristine LaCa-LDH, CeCa-LDH, and CS. The two rare earth-based LDH/CS can remove 96% of phosphate from natural water, and the removal performances remained over 95% after five cycles of sorption [41].

**Figure 3:**
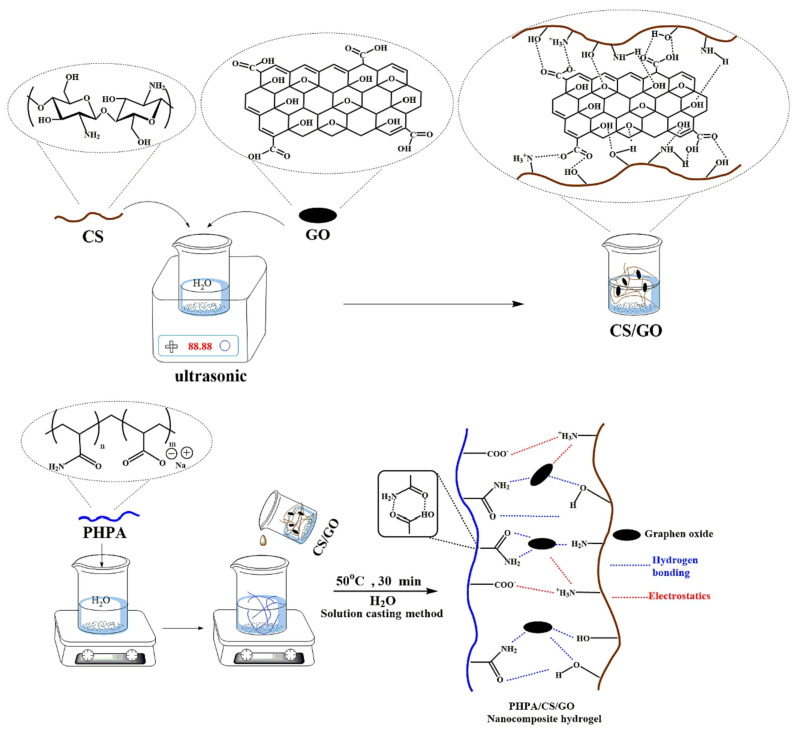
Schematization of the self-assembly of nanocomposite GO hydrogel in a chitosan (brown line)/partially hydrolyzed polyacrylamide (PHPA) network (blue line). Reprinted with permission from Ref. [42]. Copyright 2023, Ali Rahmatpour, International Journal of Biological Macromolecules, Elsevier.

Besides metal nanomaterials, different carbon-based nanomaterials are successfully integrated into bio-based hydrogels, enhancing their sorption capabilities towards different contaminants. Activated carbon, graphite, graphene, carbon nanotubes, fullerenes, and polyaromatic molecules are among the carbon-based nanomaterials widely employed for composite/hybrid production applied in water remediation [80]. Graphene oxide (GO), a two-dimensional carbon allotrope with surface and edges characterized by a variety of oxygen functional groups (–OH, –O–, –COOH, diol, and so on), is widely used in adsorption for wastewater treatment [81]. Importantly, 2D graphene oxide sheets are hydrophilic, readily dispersed in water, and compatible with polymers due to their huge surface area and oxygen-containing groups on the edges and basal planes. This results in increased mechanical qualities compared to standard starting materials. Therefore, GO nanosheets may be inserted into biopolymer-derived three-dimensional hydrogel composites or combined with synthetic polymers to form nanocomposite materials with improved features, including fast adsorption equilibrium and surface area [82]. Rahmatpour et al. developed a new chitosan-based ternary nanocomposite hydrogel film by integrating graphene oxide nanosheets into a chitosan/partially hydrolyzed polyacrylamide (PHPA) network to improve adsorption effectiveness using a one-step self-assembly technique in water, in which H-bonding interactions cause the creation of a stable cross-linking network structure (Figure 3). 

GO loading improved surface roughness, swelling percentage (from 21,200% to 35,800%), elastic modulus (up to 73.7 Pa), and adsorption properties (from 282 mg/g to 468 mg/g). The nanocomposite demonstrated excellent thermal/pH responsiveness characteristics. The batch adsorption studies assessed the methylene blue (MB) adsorption performance of the hydrogel nanocomposite. After 45 min, MB adsorption equilibrium was attained, with an adsorption capacity of 476.19 mg·g^−1^ at an initial concentration of 100 mg/L [42].

Carbon dots (CDs) have lately gained popularity due to their distinctive optical features, low toxicity, ease of synthesis, availability, and low-cost precursors. Despite the many synthesis techniques for obtaining carbon dots (CDs), bottom-up approaches remain the most extensively used route for achieving large-scale and low-cost synthesis [83]. In addition to their known uses in bioimaging, photocatalysis, and biochemical sensing, CDs are likely to have unique applications in several environmental sectors. Compared to other carbon-based nanomaterials, CDs unique nanostructures and characteristics may allow for superior environmental capabilities in environmental sensing, contaminant sorption, membrane separation, photocatalytic degradation, and as an antibacterial agent [84]. In regard to their photocatalytic performances, the inclusion of CDs improves the features of photocatalytic systems owing to their up-conversion and photoinduced electron properties and efficient electron–hole separation. The application of CDs in the photocatalytic degradation of pharmaceuticals, dyes, and generally in water treatment is deeply analyzed by researchers [85,86].

In this regard, Seema et al. developed a novel water purification technique based on a composite material, including carbon dots (CDs) contained within a porous hydrogel. In particular, a chitosan/carboxymethyl cellulose hydrogel containing CDs (epichlorohydrin was added as a cross-linking agent) was developed as a versatile and efficient water purification platform. Embedded CDs improve light absorption throughout a wide spectral range, allowing for effective photothermal heating and consequent evaporation of gel-incorporated water. The CDs/hydrogel composite is simple to make using readily available and environmentally friendly reagents.

It is significantly less expensive than systems that use noble metals for photothermal conversion, as well as energy-intensive traditional techniques like reverse osmosis and thermal evaporation. Despite many purification processes, the CDs/gel composite remained stable, recyclable, and robust. Moreover, the CDs/hydrogel composite was used for a variety of water treatment applications, including desalination and the removal of heavy metal ions, detergents, and organic compounds from polluted water. The concentration of Cu^2+^, Ni^2+^, Ag^+^, and Cd^2+^ in water removed by a CDs-20/gel before illumination at 1 kW/m^2^ intensity for 1 h was 0.01 M. Interestingly, after illumination, less than 0.05% of the original ion concentrations were found in the recovered water **[43]**.

#### 2.1.2. Clay-Doped Bio-Based Hydrogels for Pollutant Sorption

Clay–hydrogel nanocomposites, a promising class of materials for environmental applications, is gaining attention for their potential to effectively remove pollutants from wastewater due to their exceptional adsorption capabilities, ecological compatibility, customizability, and enhanced reusability. Clays are nanostructured, fine-grained minerals that can be classified according to their mineral content, size, and structure into different classes, such as montmorillonite, kaolinite, illite, bentonite, and chlorite [87]. Clays possess extensive surface area, notable swelling capacity, particularly montmorillonite and bentonite clays, cation exchange capability, and substantial adsorption/absorption abilities due to their negatively charged surface. The surface chemistry of clays determines their physical and chemical characteristics, influencing their adsorption capabilities [57]. Clay minerals have demonstrated a great affinity for metals, organic dyes, and emerging pollutants and can be utilized as is, without alteration, successfully. Pre-treatment can increase both pollutant selectivity and clay mineral adsorption capabilities [57,88]. 

These physicochemical modifications of clays resulted in an increase in pore volume, surface area, and many spots on the surface of clays that rendered them organophilic and hydrophobic, hence enhancing the absorption of organic and inorganic pollutants. The presence of various functional groups (–OH, –COOH, and –NH_2_) along the polymer chain, combined with the adsorption properties of clays, renders these nanocomposites effective at removing a wide range of contaminants from various sources [89,90].

Researchers have extensively studied the absorption mechanism of pollutants on clay–hydrogel nanocomposites, discovering that electrostatic attraction, hydrogen bonding, and complexation are the most powerful methods for capturing and removing pollutants such as dyes and metals from contaminated media [55].

Several synthetic routes have been used to create clay–hydrogel nanocomposites, including free radical polymerization, supramolecular assembly, freeze-thawing, and grafting [55]. Polymers formed by chemical cross-linking are commonly desired due to their features such as tensile strength, swelling, stiffness, and permeability.

In this regard, Pal et al. developed a gelatin–bentonite composite (GBC) gel with or without bis-acrylamide and formaldehyde as cross-linkers that were utilized as adsorbents to remove lead (II) from aqueous solutions. The testing results revealed that GBC has a maximum adsorption capacity of 47.169 mg/g at 25 °C towards Pb^2+^. The desorption experiment with 0.1 N nitric acid demonstrated that the effect of repeated use is great and that the used adsorbents may be totally recovered [44].

**Figure 4:**
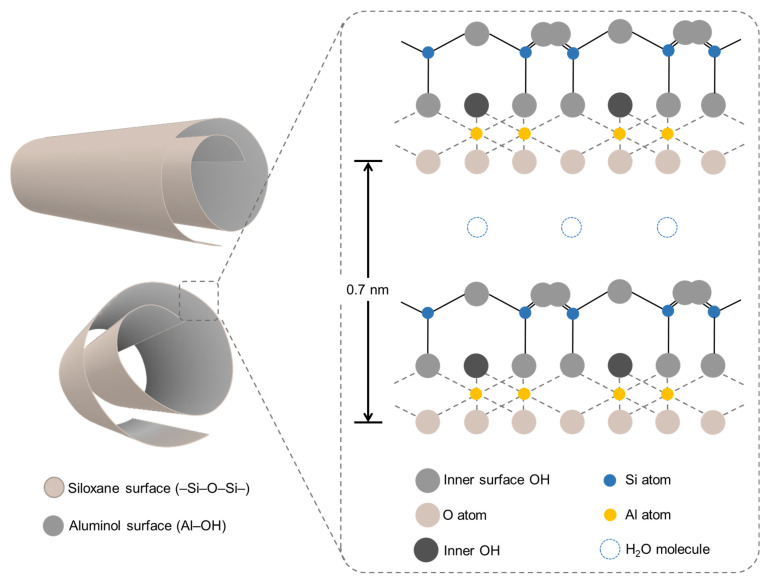
Illustration of the composition of halloysite clay nanotubes.

Other approaches can include the formation of hydrogel beads using proper biopolymers and coagulant agents and including different functional clays, such as halloysite, as functional nanofillers as efficient sorbent systems for different pollutants. Kaolinite and halloysite are the two most extensively researched and utilized aluminosilicates with the identical chemical composition (Al_2_Si_2_O_5_(OH)_4_) with alternating octahedral alumina (AlO_6_) and tetrahedral silica (SiO_4_) sheets organized in a 1:1 ratio. The primary distinction between kaolinite and halloysite is their shape. Kaolinite occurs naturally as platelet sheets, whereas halloysite is composed of rolled sheets organized to form tubular particles [91] (Figure 4). 

Halloysite deposits have been discovered in numerous countries around the globe, such as China, New Zealand, Brazil, the United States, France, Algeria, and Turkey. These deposits are often found alongside a mix of different clay minerals, quartz, feldspars, and oxides in different proportions. Halloysite clay is extensively utilized by ceramic industries worldwide for creating both traditional and cutting-edge ceramics due to its elevated Al_2_O_3_ content (>30 wt%), malleability, whiteness, and non-toxic characteristics [92]. Extensive research is being conducted on this material due to its biocompatibility (minimal toxicity in both in vivo and in vitro settings) along with its beneficial mechanical, physical, and chemical characteristics. These studies aim to explore the potential applications of this material in various industries for the development of products such as polymers, cosmetics, medical supplies, agricultural materials, nanofillers, catalyst support materials, nanocontainers, adsorbents for hazardous metals, anticorrosion agents, and more.

Due to its tubular structure at the nanoscale, this material can be filled with different substances for the controlled release of various active agents, including antioxidants [93], flame retardants [94,95], corrosion inhibitors [96], biocides, and pharmaceuticals [97]. Additionally, halloysite can be modified with organosilanes through straightforward methods like sol–gel synthesis to improve its dispersion within polymer blends, enhance thermal stability and tensile strength, and create hydrophobic nanocomposites suitable for a wide range of uses [98,99,100,101,102]. 

Viscusi et al. made up a green, unique, and sustainable technology for efficiently removing methylene blue from aqueous solutions employing halloysite as a nanofiller. In particular, the suggested system was developed starting with a sodium alginate/soybean extract blend strengthened with hemp hurd derived from agricultural waste and halloysite nanotubes. Composite beads were easily made using the ionotropic gelation process, which used calcium chloride as a cross-linking agent. The study found that adding 35% wt of halloysite nanotubes increased the adsorption capacity of methylene blue from 32 to 49 mg/g by increasing the inorganic filler content [45]. 

A high-performance magnetic bio-based κ-carrageenan/kaolinite/Al_2_O_3_/Fe_3_O_4_ composite hydrogel (CKAlFe) was meanwhile developed by Shahinpour et al. and characterized and utilized to adsorb both Alizarin Red S and Congo red dyes from an aqueous solution. Alumina-coated iron oxide core–shell nanoparticles were employed as the composite magnetic component to increase its chemical stability and create additional adsorption sites with specialized functions. The coating of magnetic particles with a highly porous polysaccharide and clay provides potential paths for target adsorption in the diffusing medium and aids composite separation by placing an external magnetic field. Morphological analysis revealed the successful synthesis of the non-rigid plate-like CKAlFe adsorbent with a microporous structure, where κ-carrageenan forms an amorphous component and kaolinite clay, Al_2_O_3_, and Fe_3_O_4_ comprise the crystalline portion. This adsorbent was found to be highly effective, demonstrating maximum adsorption capacities of 26.9 mg/g for Congo red and 33.5 mg/g for Alizarin Red S molecules from binary solutions [46]. 

**Figure 5:**
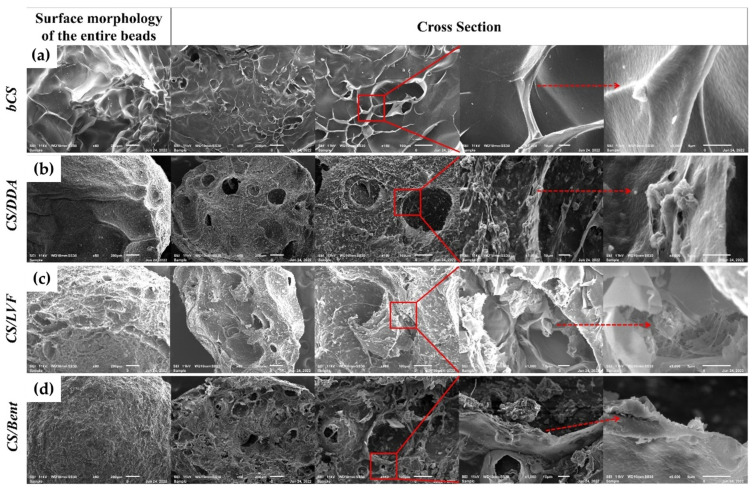
SEM micrographs showing chitosan-based beads (**a**) doped with dodecylamine (**b**), dellite LVF (**c**), and bentonite (**d**); red squares and arrows represent the zoomed area showed in the respective right side image Reprinted with permission from Ref. [47]. Copyright 2022, Estefanía Baigorria, Journal of Water Process Engineering, Elsevier.

Hybrid chitosan/clay nanocomposite hydrogels as possible paraquat biosorbents in water were also developed by Baigorria et al. The materials were synthesized using basic procedures, yielding nanocomposite beads with semi-spherical symmetry and micrometric sizes. The physicochemical and morphological characterization revealed that the presence of nanoreinforcements causes an interaction with the chitosan biopolymeric matrix, significantly altering its structure and characteristics. The beads demonstrated a high level of porosity with interconnected pores, as shown in the SEM image of Figure 5. Paraquat aqueous sorption studies on dodecylamine (DDA)-, dellite LVF (LVF)-, and bentonite (Bent)-modified chitosan nanocomposite beads produced good results, showing maximum adsorption capacities of 0.98, 0.94, and 0.99 mg·g^−1^, respectively. Furthermore, these experiments demonstrated that the addition of nanoclays to chitosan matrices is required for efficient paraquat biosorption [47].

#### 2.1.3. Lignocellulosic- and Cellulosic-Doped Hydrogels for Pollutant Sorption

Hydrogels based on biopolymers, such as cellulose, exhibit several benefits because of their enhanced functionality, larger surface area, affordability, greater adsorption capacity, biodegradability, and ease of manufacture and recycling. Moreover, the remarkable hydrophilicity of cellulose-based hydrogels makes them a feasible adsorbent for the treatment of wastewater. According to recent research studies, various types of cellulose materials are currently used in the production of hydrogels, such as bacterial and native/pure cellulose, as well as their derivatives (e.g., ester derivatives like acetate succinate, acetate trimellitate, hydroxypropyl methyl phthalate, hydroxypropyl methyl phthalate, etc.) and/or their composites [103]. 

On the other hand, the employment of cellulose, cellulose derivatives, or cellulosic compounds as fillers and functional agents in polymeric blends for hydrogel development represents a solution to the challenges related to the low solubility of native/pure cellulose. In this regard, various cellulose derivatives (i.e., micro-nano-cellulose, cellulose micro-nanocrystals, micro-nano-fibrillated cellulose, and bacterial micro-nano-cellulose) can be easily synthesized via chemical or enzymatic methods and employed for these purposes [104]. 

**Figure 6:**
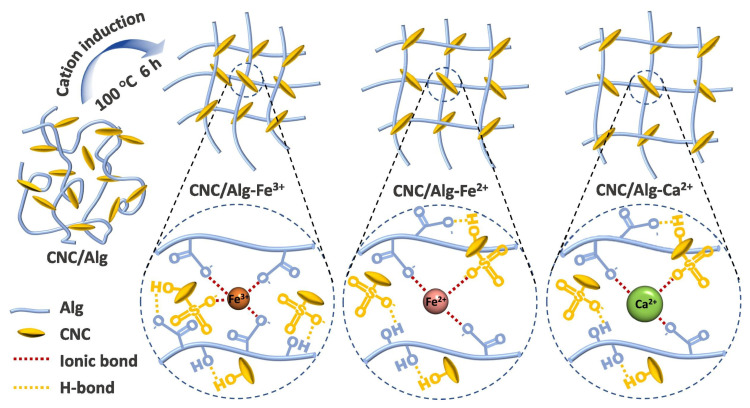
Formation of hierarchical hydrogel scaffolds of alginate reinforced with cellulose nanocrystals induced using varied cations (Fe^3+^, Fe^2+^, and Ca^2+^). Reprinted with permission from Ref. [48]. Copyright 2024, Luna Tie, Separation and Purification Technology, Elsevier.

For example, Tie et al. developed a bio-based alginate reinforced with cellulose nanocrystal hydrogel via Fe^2+^ cross-linking (CNC/Alg-Fe^2+^) and low-temperature heat treatment (100 °C) and employed it as an excellent adsorbent for tetracycline (TC) removal. Metal ions (Fe^3+^, Fe^2+^, and Ca^2+^) allowed binds with hydroxyl, sulfate half ester, and carboxyl groups of the CNC/Alg surface through chelation, strong hydrogen bonds, and electrostatic interactions (Figure 6). The hydrogel cross-linked with Fe^2+^ had the highest adsorption capacity of TC (741.66 mg·g^−1^), which was approximately 1.9 and 6.9 times higher than that of the hydrogel cross-linked with Fe^3+^ (394.16 mg·g^−1^) and Ca^2+^ (108.14 mg·g^−1^) due to enhanced chelation between Fe^2+^ and TC (at 298 K). The synergistic effect of surface complexation, cation bridging, n-π electronic donator–acceptor interactions, hydrogen bonding, and electrostatic interactions allowed for the efficient sorption of TC. Furthermore, the adsorption capacity of CNC/Alg-Fe^2+^ remained 84.6% after five regeneration cycles, showing strong reusability and good features for the purification of TC-containing biomedical wastewater [48].

Bora et al. demonstrated the eco-friendly and non-toxic fabrication of a nanohybrid utilizing modified cellulose nanofiber and zinc oxide nanoparticles, which was then mixed into a starch/itaconic acid/acrylic acid ternary hydrogel matrix. The resultant biodegradable hydrogel nanocomposites were used to remove metal ions (i.e., Cu(II) and Fe(II) from an aqueous solution), demonstrating excellent adsorption capacity. In particular, the maximum water swelling capacity of 580 g/g was observed after including 0.25 wt% nanohybrid. 

Furthermore, the addition of the nanohybrid improved the hydrogel nanocomposites metal ion adsorption ability, resulting in removal capacities of 122 and 70 mg/g for Cu(II) and Fe(II), respectively. The nano-structured hybrid adsorbent was simply removed and reused for three purification cycles without losing efficiency [49].

Lignin is the most abundant and only scalable renewable aromatic resource on the planet. It has been produced as a significant waste from the pulp sector (70 million tonnes per year), and more than 95% of the industrial lignin is not fully used. Because of their unique physicochemical qualities, such as high carbon content, abundant functional groups, and environmental friendliness, lignin-based porous materials have demonstrated encouraging results in the effective removal of soluble contaminants from wastewater. The most common lignin-derived porous materials include hydrogels, aerogels, porous carbon, and other porous materials. These materials maintain their original structural and surface chemical characteristics [105]. This resource was employed by Liu et al. to remove aqueous Cr(VI). In this regard, a lignin hydrogel-supported nZVI (nZVI@LH) was produced using various precursor Fe(II) ion concentrations. Zero-valent iron nanoparticles (NZVI) are commonly used in groundwater remediation due to their high reactivity to a wide range of contaminants [106,107]. In contrast, NZVI particles are prone to aggregation, oxidation, and sedimentation. Surfactants, suspending agents, and stabilizers are commonly used to address these issues [108]. Using lignin hydrogel to support iron is an effective solution for cleaning up the environment. Supporting nZVI with the polymer is a new strategy that can lower the danger of nanoparticle release while also improving its reactivity. For the development of the nZVI@LH composite, nZVI particles were attached to the surface of lignin hydrogel through the Intermatrix Synthesis (IMS) method. It was shown that lignin hydrogel served as an effective support for nZVI particles, resulting in the well-distribution and stability of highly reactive nZVI particles. Moreover, nZVI in lignin hydrogel demonstrated a better Cr(VI) removal capability than pure nZVI [50].

The discovery of biochar has created a hotspot in several research sectors, including agriculture, energy, environment, and materials. Biochar-based products provide an innovative solution to environmentally problematic challenges [109]. Currently, biochar is gaining popularity as a renewable, efficient, ecologically friendly, economical, and green biomaterial for environmental remediation purposes. Biochar is a carbon-rich pyrolysis product derived from a variety of biomass sources, typically vegetal or animal waste, with the source type and pyrolysis circumstances determining its physical and chemical properties. The abundance of surface functional groups (e.g., hydroxyl, carboxyl, and amino), high porosity, wide surface area, and superior ion exchange capacity all contribute to biochar-based adsorbents very high adsorption effectiveness. In addition to its excellent adsorption efficiency, biochar has the following advantages: sustainability, ease of manufacture, functionalization, stability, improved physicochemical qualities, and recyclability [110].

**Figure 7:**
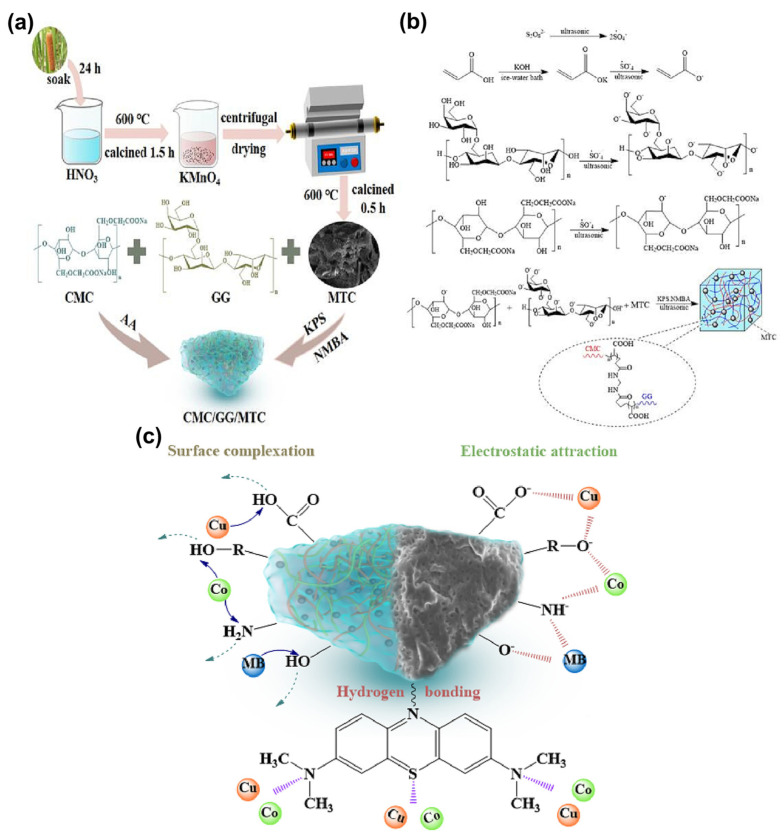
Schematization of MTC and CMC/GG/MTC adsorbent production (**a**), CMC/GG/MTC synthesis reaction scheme (**b**), and potential interaction of the composite hydrogel sorbent with Cu^2+^, Co^2+^, and MB (**c**). Reprinted with permission from Ref. [51]. Copyright 2023, Lingze Yang, International Journal of Biological Macromolecules, Elsevier.

Thanks to these advantages, biochar is effectively inserted into hydrogel matrices to obtain functional hydrogel composites. In these regards, Yang et al. employed *Typha angustifolia* as a charcoal source and altered it using a powerful oxidizing agent, potassium permanganate (KMnO_4_), to produce modified *Typha angustifolia* (MTC). The green, stable, and efficient CMC/GG/MTC composite hydrogel was effectively synthesized by free radical polymerization of MTC employing carboxymethyl cellulose (CMC) and guar gum (GG) (Figure 7a–c).

The experimental findings revealed that the adsorbent had a good adsorption effect towards Cu^2+^, Co^2+^, and MB with adsorption capacities of 805.45 mg·g^−1^, 772.52 mg·g^−1^, and 598.28 mg·g^−1^, respectively. Moreover, the sorbent maintained a good adsorption capacity after five rounds of testing [51].

In another case, Kamel et al. developed magnetic hydrogels made from cross-linked carboxymethyl cellulose grafted acrylamide (CMC-g-AM) embedded with porous carbon (PC) and citric acid-modified magnetite. PC was synthesized by oxidizing bagasse in a single step under muffled atmospheric conditions. Magnetite (Fe_3_O_4_) nanoparticles were synthesized via co-precipitation (Fe^2+^/Fe^3+^) and citric acid modification (CFe). 

Because of the particular role of the cross-linked CMC matrix in fostering synergy between embedded PC and CFe, magnetic hydrogels demonstrated the ability to remove Pb-ions and methylene blue dye (MB) from water with great efficacy. Adsorption tests using time intervals (5–120 min) and Pb-ions and MB concentrations (5–500 mg/L) showed that CMC-g-AM containing equal amounts of PC and CFe had significantly higher removal efficiency: 70.8 and 96.1% compared to 47.8 and 30.2% (without PC and CFe) for Pb-ions and MB adsorption, respectively. 

In particular, the results showed that the hydrogel had the highest adsorption capacity of 294.11 and 222.2 mg/g for Pb-ions and MB, respectively, compared to the poor sorption capacity of the unmodified CMC-g-AM hydrogel [52].

## 3. Stimuli-Responsive Hydrogels for (Waste) Water Monitoring

### 3.1. Stimuli-Responsive Hydrogels: Design and Development

Traditional sensor technologies face challenges such as limited selectivity, stability, and environmental sustainability. In this regard, bio-based polymeric hydrogels offer a promising alternative for wastewater sensor applications due to their biocompatibility, tunable properties, and eco-friendliness [111,112].

The concept of stimuli-responsive hydrogels dates back to the mid-20th century, with early research focusing on the synthesis and characterization of hydrogels that could respond to changes in environmental conditions. Early examples include temperature-responsive hydrogels based on poly(N-isopropylacrylamide) (PNIPAAm), which undergo a reversible phase transition near physiological temperature, leading to changes in swelling behavior and drug release kinetics [113,114].

The development of stimuli-responsive bio-based hydrogels represents a significant advancement in the field of biomaterials and smart materials. They are designed to undergo reversible changes in their structure, properties, and functionality in response to specific external stimuli, such as variations in temperature, pH, light, electric field, or the presence of certain ions or molecules. This responsiveness enables a wide range of applications such as drug delivery, tissue engineering, biosensing, and smart devices. Thus, the development of stimuli-responsive hydrogels has evolved through various stages, driven by advances in polymer chemistry, material science, and biomedical engineering [115,116], enabling the synthesis of novel monomers and polymers with tailored responsiveness to specific stimuli. 

In this regard, researchers have explored a wide range of monomers, including acrylic acids, acrylamides, vinyl ethers, and others, to design hydrogels with desired responsiveness to temperature, pH, light, and other stimuli [117]. Polymerization techniques such as free radical polymerization, reversible addition-fragmentation chain transfer (RAFT) polymerization, and controlled/living polymerization have been employed to control the molecular architecture and properties of stimuli-responsive hydrogels [118]. Lu et al. developed stimuli-responsive sodium alginate beads with controllable swelling behavior, pH sensitivity, and high adsorption capacity via a post-cross-linking method in large-scale production [111].

#### 3.1.1. Stimuli-Responsive Hydrogels Synthetic Approaches

Designing stimuli-responsive hydrogels involves careful selection of polymer chemistry, cross-linking methods, and incorporation of responsive moieties. Their synthesis uses a mix of polymerization procedures, cross-linking methods, and post-polymerization changes to generate tailored responses to certain external stimuli. The design approaches and synthesis techniques frequently used to create stimuli-responsive hydrogels are listed as follows:

1. Polymer selection:
  - Responsive polymers, either select responsive polymers, such as thermoresponsive polymers like poly(N-isopropylacrylamide) (PNIPAAm) or pH-responsive polymers like poly(acrylic acid) (PAA), that have a natural capacity to respond to particular stimuli. Responses to external stimuli cause these polymers to undergo conformational changes, which alter the features of the hydrogel [119,120].
  - Smart copolymers are opportunely developed copolymers that are both sensitive and inert in order to attain structural integrity and stimuli response. Block copolymers and graft copolymers, for instance, can combine responsive and non-responsive portions to produce tailored responsiveness. For example, copolymerizing N-isopropylacrylamide (NIPAAm) with hydrophilic monomers such as acrylic acid (AAc) or N,N-dimethylacrylamide (DMAAm) can yield thermoresponsive hydrogels with tunable swelling behavior [121,122].
  - Responsive functional groups based on incorporated responsive functional groups, like pH-responsive or thermoresponsive moieties, into the side chains or backbone of the polymer. Responses to external stimuli cause these functional groups to undergo conformational modifications or protonation/deprotonation, leading to changes in hydrogel properties [123,124].
2. Cross-linking methods:
  - Chemical cross-linking, obtained by techniques such as free radical polymerization or Michael addition, can be used to form covalent connections between polymeric chains. These cross-linking events keep the hydrogel network stable while also allowing it to respond to stimuli. For instance, introducing temperature-sensitive cross-linkers such as N,N’-methylenebis(acrylamide) (MBAAm) into the polymerization mixture can produce thermoresponsive hydrogels [125,126].
  - Physical cross-linking is related to reversible bonds between polymer chains obtained by using physical cross-linking techniques such as hydrogen bonding, hydrophobic interactions, or host-guest interactions. Physical cross-linking enables stimulus-responsive behavior while preserving reversibility and dynamic features [127]. For example, incorporating host-guest complexes, such as cyclodextrin-encapsulated guest molecules, into hydrogel matrices can yield pH-responsive hydrogels capable of reversible swelling and deswelling behavior [128,129].
  - Stimuli-responsive hydrogels can also be tailor-made for a variety of uses in drug administration, tissue engineering, sensing, and smart materials by carefully choosing the synthetic approach and the hydrogel starting reactants.

#### 3.1.2. Stimuli-Responsive Mechanisms

By carefully choosing polymers and functional groups, scientists can design hydrogels to respond to specific stimuli with desired effects. Stimuli-responsive mechanisms refer to the underlying processes by which hydrogels undergo reversible changes in their structure, properties, or behavior in response to specific external stimuli. In the material world, stimuli-responsive hydrogels are similar to chameleons in that they change their characteristics in response to outside stimuli [130,131,132].

These stimuli can include changes in temperature, pH, light, ionic strength, or the presence of certain molecules, thanks to different stimuli-responsive mechanisms observed in hydrogels.

##### Thermoresponsive Mechanism

Thermoresponsive hydrogels are an interesting family of hydrogels that may alter their characteristics in reaction to temperature fluctuations. They show changes in swelling behavior or sol–gel transition in response to temperature differences [133,134,135].

These hydrogels are formed from specially designed polymers that contain functional groups that become more or less water-soluble (hydrophobic) depending on the temperature. They are crafted with special functional groups that exhibit temperature-dependent water solubility. At low temperatures, these groups become hydrophilic (water-loving), and at high temperatures, they become hydrophobic (water-repelling). Thus, water molecules play a crucial role in determining the hydrogel structure and responsiveness. In fact, they interact with the functional groups on the polymers, influencing the overall organization of the hydrogel network. At low temperatures (below the Lower Critical Solution Temperature, or LCST), the functional groups on the polymers are hydrophilic. This strong attraction between the functional groups and water molecules causes the polymer chains to extend outwards, forming a loose network with spaces filled by water, resulting in a swollen hydrogel. As the temperature rises above the LCST, the functional groups on the polymers undergo a transformation. They become more hydrophobic, reducing their affinity for water. This weakens the attraction between the polymer chains and water molecules. Due to the decreased water love, the polymer chains start to collapse and clump together to minimize their contact with water. This aggregation process leads to a denser network with less space for water molecules. Consequently, the swollen hydrogel starts to expel water trapped within its network. This expulsion of water causes the hydrogel to shrink and become more compact [136,137]. 

The thermoresponsive mechanism offers a versatile approach for designing hydrogels with tailored properties and functionalities. The most widely studied thermoresponsive polymer is poly(N-isopropylacrylamide) (PNIPAAm), which undergoes a lower critical solution temperature (LCST) phase transition around 32 °C. Below the LCST, PNIPAAm hydrogels are hydrated and swollen, while above the LCST, they collapse and expel water due to hydrophobic interactions. The PNIPAM hydrogel made by Liu et al. [138] is another illustration of a thermally sensitive hydrogel. Their unique recovery hysteresis during cooling distinguished the PNIPAM hydrogel they produced, which had a smooth thermoresponse during heating. Moreover, a thermoresponsive PNIPAM hydrogel was created by Kim et al. The swelling behavior of PNIPAM-PEG hydrogels may be efficiently controlled by varying the molecular weight and concentration of the polyethylene glycol (PEG) cross-linker. A portion of the current hydrogels that respond to heat are created using surface-modified PNIPAM groups [139,140,141,142]. This paves the way for innovative applications in medicine, biotechnology, and beyond.

##### pH-Responsive Mechanism

In contrast to thermoresponsive hydrogels that react to temperature, pH-responsive hydrogels depend on the strength of acidity or alkalinity and alter their structural conformation or swelling behavior in response to pH variations [143,144,145]. This provides an additional fascinating mechanism for these materials to adjust to their surroundings. 

For example, hydrogels containing pH-sensitive functional groups such as carboxylic acids (–COOH) or amino groups (–NH_2_) can undergo protonation or deprotonation in response to acidic or alkaline environments, leading to changes in charge density and hydrogel swelling [146,147,148,149]. These functional groups can act like tiny magnets with positive or negative charges [150,151,152].

PDMAEMA/crystalline nanocellulose (CNC) hydrogels were utilized to extract methyl orange (MO) from water through the use of intermolecular and electrostatic interactions between the functional groups of the hydrogel and dye [151].

To adsorb and desorb anionic dyes at various pH levels, a hydrogel with an interpenetrating network of carboxymethyl cellulose and chitosan was created by Wang et al. Under acidic conditions, the amino functional groups of chitosan were protonated to create –NH_3_^+^. Electrostatic interactions allow the protonated amino groups on the chitosan surface to absorb negatively charged Acid Orange II. Furthermore, under alkaline conditions, the amino groups on the hydrogel were retained in the form of –NH_2_, therefore desorbing the dyes into the system [153]. Ever the reliable partner, water molecules play a crucial role in influencing the hydrogel structure and response. In fact, they can interact with the charged functional groups, affecting how the polymer chains arrange themselves. In detail, the pH level of the surrounding environment determines the initial charge state of the functional groups on the polymers. At a specific pH, the functional groups on the polymer chains might have similar charges. These like charges repel each other, forcing the polymer chains apart. Imagine the polymer chains pushing away from each other like charged balloons. Due to this repulsion, the polymer chains spread out, forming a more open and expanded network within the hydrogel. This expanded network creates more space for water molecules to reside. 

As a consequence of the expanded network, the hydrogel absorbs more water and swells. This swelling can be quite significant, depending on the design of the hydrogel. In a different pH environment, the functional groups on the polymer chains might acquire opposite charges. These opposite charges attract each other, pulling the polymer chains closer together. Due to the attractive forces between the oppositely charged functional groups, the polymer chains collapse and form a denser network with less space for water molecules. Consequently, the swollen hydrogel starts to expel water trapped within its network. This expulsion of water causes the hydrogel to shrink and become more compact. By tailoring the functional groups and their pKa (acid dissociation constant), scientists can create pH-responsive hydrogels for various and innovative applications in medicine, agriculture, environmental monitoring, and more [154,155].

##### Light-Responsive Mechanism

Light-responsive hydrogels, in contrast to temperature or pH, use light as a trigger, providing exact control and spatial manipulation. Photo-responsive moieties, such as azobenzene, spiropyran, or o-nitrobenzyl groups, can undergo reversible photoisomerization or photolysis upon exposure to specific wavelengths of light, triggering changes in hydrogel structure and properties such as swelling, degradation, or mechanical strength. Typically, they consist of two components: long-chain polymers, which serve as the structural backbone of the hydrogel and form a network that traps water, and photo-responsive molecules, which are integrated into the hydrogel network and function like small switches that flip when exposed to particular wavelengths of light [156,157].

The photo-responsive molecules are either attached to these polymers or integrated within the network itself, and they respond chemically, sometimes breaking bonds, sometimes altering shape, and sometimes producing heat when the proper wavelength of light is applied to them. Photo-responsive molecules interact with the surrounding polymer network in a different way due to the chemical reaction that is induced by light. There are several options that might be involved here. On one hand, the light might cause the photo-responsive molecules to break apart, disrupting the cross-links holding the polymer chains together. This leads to a looser network with more space for water, causing the swelling of the hydrogel. On the other hand, light exposure might induce the photo-responsive molecules to change their shape, and this can alter their spacing within the network, influencing how the polymer chains arrange themselves. Furthermore, sometimes certain photo-responsive molecules can convert light energy into heat. A temperature-responsive mechanism within the hydrogel may then be activated by this localized heating.

By carefully selecting photo-responsive molecules, it is possible to design hydrogels with tailored functionalities triggered by light. This opens exciting avenues for applications in medicine, biotechnology, and advanced material development.

In detail, numerous studies indicate that light-responsive polymers are effective adsorbents in wastewater treatment. The construction of an eco-friendly, photo-responsive supramolecular polysaccharide hybrid hydrogels able to promote plant growth and synchronous capture of heavy metal ions from arylazopyrazole-modified hyaluronic acid (HA-AAP), guanidinium-functionalized β-cyclodextrin (Guano-CD), and laponite clay (LP) was reported by Y.-H. Zhang et al. After releasing the cargo, the hydrogels could be used to capture heavy metal ions via strong complexation between the ions and carboxyl groups [158].

Because light-responsive devices allow for spatio-temporal control of drug release, they are also appealing as drug delivery carriers [159].

An agarose hydrogel matrix containing gold nanoparticles (AuNPs) covered with polymer (poly(methacryloxyethyl trimethyl ammonium chloride) [P(METAC)]) was used by Basuki et al. to create a visible light-controlled protein delivery system. The AuNPs converted photons to heat (thermal energy), leading to a localized temperature increase (>45 °C) in the agarose hydrogel under irradiation with visible light. Therefore, with the release of loaded drugs, the agarose matrix with a low melting point (Tm > 45 °C) underwent reversible softening. The author provided evidence that the AuNP/hydrogel system activated by visible light has the ability to regulate the release profile by varying the quantities of AuNPs and agarose, as well as the strength and duration of the light [160,161].

##### Redox-Responsive Mechanism

In the realm of stimuli-responsive hydrogels, the redox-responsive mechanism offers a unique way for these materials to interact with their environment. Unlike temperature, pH, or light, the redox-responsive mechanism offers a sophisticated way to design hydrogels that interact with the cellular or environmental redox state [162,163]. Because of their high water retention capacity and unique structure for controlled release, redox-responsive hydrogels have been widely studied as carriers for drug delivery and tissue engineering applications [164]. 

Redox-sensitive linkages are the essential components, acting like chemical hinges within the hydrogel network, and they are designed to be susceptible to reduction (gaining electrons) or oxidation (losing electrons), while polymers always represent the backbone of the hydrogel, with their long-chain molecules that are often linked together by the redox-sensitive linkages. They can also incorporate some redox species that trigger the response [165,166], or they can be oxidizing agents (causing oxidation) or reducing agents (causing reduction), depending on the design of the hydrogel. 

Common redox species include glutathione (a reducing agent found in cells) and reactive oxygen species (ROS, oxidizing agents produced by cells) [167,168]. The initial oxidation state of the redox-sensitive linkages determines the structure of the hydrogel, until a redox species (oxidant or reductant) comes into contact with the hydrogel and interacts with the redox-sensitive linkages. The interaction between the redox species and the linkages causes them to undergo a chemical change that can involve either the break within the linkages, disrupting the cross-links that hold the polymer chains together, or a change in oxidation state that might alter the way the linkages interact with each other or with water molecules. 

Y. Li et al. effectively synthesized a redox-responsive and green-based hydrogel from activated carboxymethyl cellulose nanocrystals and L-cysteine. The resultant disulfide cross-linked hydrogel may regulate the adsorption or release of dye, such as methylene blue (MB), by responding quickly to external oxidizing or reducing stimuli [163].

##### Molecular Recognition-Responsive Mechanism 

Molecular recognition-responsive hydrogels are a fascinating class of stimuli-responsive hydrogels that take the concept of responsiveness to a whole new level. Unlike traditional stimuli like temperature or pH, this kind of hydrogel responds to the presence of specific molecules, offering highly selective and targeted responses [169]. For example, hydrogels containing host-guest complexes can undergo conformational changes or binding events in response to the presence of target molecules, enabling applications in biosensing, drug delivery, and molecular capture [170,171,172]. For this reason, they need to include:

- Receptor molecules act like sophisticated locks within the hydrogel network. The receptor molecules are often attached to or integrated within the polymer chains. The receptor molecules within the hydrogel are tailored to recognize and bind only to the target molecules, which ensures a highly selective response.
- Target molecules are the external players that trigger the response. They are the specific molecules the receptor molecules are designed to recognize and bind with.

In detail, when a target molecule encounters a receptor molecule in the hydrogel network, they specifically bind together like a key fitting into a lock. This binding event can trigger various changes within the hydrogel. The specific response of the hydrogel depends on the nature of the binding interaction and the design of the system, and it can involve the swelling/deswelling depending on the changes in cross-linking or interactions within the network, the cargo release if the hydrogel encapsulates other molecules, or some fluorescence change if they incorporate fluorescent molecules that change their properties upon binding to the target, allowing for detection purposes [173].

In particular, depending on the design of the hydrogel, the binding event can lead to several possibilities:

- The binding of target molecules can act as additional cross-linking points within the network, causing the hydrogel to become denser and potentially expel water, leading to shrinkage.
- The receptor molecules might undergo a conformational change upon binding to the target, altering their interactions with the surrounding polymer network or water molecules. This can influence the hydrogel’s swelling or stiffness.
- In some cases, the binding event might trigger the enzymatic degradation of the hydrogel network, leading to its breakdown.

Using the molecular recognition-responsive process, hydrogels with unmatched selectivity and responsiveness may be designed in a flexible and effective manner. This creates new and interesting opportunities for sophisticated biosensing and tailored medication delivery. J. Wan et al. demonstrated the development of a new adsorbent by the construction of a composite scavenger with a highly porous structure, giving aptamers the ability to create special, complex binding pockets that trap contaminants and provide ideal selectivity. In detail, they strategically combined the benefits of anisotropic Fe_3_O_4_–Ag Janus hybrid nanoparticles, the specific recognition abilities of aptamers, and the 3D porous structure of a cellulose-based hydrogel in a stepwise approach to develop a unique adsorbent scavenger [174].

Therefore, the understanding of stimuli-responsive mechanisms in hydrogels is a powerful tool for scientists and engineers. It allows them to create these fascinating materials with tailor-made properties that respond to specific external cues. This field holds immense potential for revolutionizing various sectors, from medicine and biotechnology to environmental monitoring and advanced material development. Table 2 summarizes the described approaches regarding the development of stimuli-responsive hydrogels with their response mechanism towards specific stimuli.

The most significant studies on eco-friendly stimuli-responsive polymer-based hydrogels, including their stimulus, mechanism, and application in the removal of contaminants, will be discussed in the next paragraphs. 

### 3.2. Applications of Bio-Based and Stimuli-Responsive Hydrogels in (Waste) Water Monitoring

By combining the benefits of bio-based materials with the power of stimuli-responsive mechanisms, scientists can create a new generation of wastewater sensors that are sustainable, efficient, and capable of providing real-time data for improved water quality management [175,176]. In particular, by carefully selecting the stimuli-responsive mechanism and tailoring the hydrogel properties, scientists can create sensors with high selectivity and sensitivity for detecting specific contaminants relevant to wastewater management. This targeted approach allows for real-time monitoring and improved control over wastewater treatment processes [177,178]. Table 3 reports some recent sustainable and bio-based stimuli-responsive hydrogels for different pollutant detection.

Stimuli-responsive mechanisms offer a powerful tool for designing bio-based hydrogels as effective wastewater sensors. In light of this, a closer examination of a few processes with significant promise for wastewater sensing applications is given hereafter.

#### 3.2.1. Heavy Metal Detection

Unlike traditional methods that require complex laboratory analysis, functionalized hydrogels offer real-time monitoring capabilities. The response of the hydrogel, whether a color change, swelling, or conductivity shift, can be observed directly, providing immediate feedback on the presence and concentration of heavy metals in the water. This real-time feedback allows for quicker decision-making and intervention strategies to address heavy metal contamination. 

Wastewater treatment plants can optimize their processes based on the sensor data, and environmental monitoring efforts can be targeted towards areas with the highest levels of contamination. By leveraging the selective adsorption and real-time monitoring capabilities of functionalized hydrogels, scientists and environmental engineers can develop effective strategies to combat heavy metal contamination in water resources, safeguarding our environment and public health, that is, a portable quantitative detector of Fe^3+^ by integrating a smartphone with the colorimetric responses of a rhodamine-functionalized polyacrylamide hydrogel chemosensor developed by Liu et al. [187].

Functionalized hydrogels exhibit selective adsorption and quantification of specific heavy metal ions, such as lead, mercury, or chromium, enabling real-time monitoring of their contamination in industrial effluents and natural water bodies. These hydrogels are specifically designed with functional groups, like ligands, that can bind strongly (chelate) with targeted heavy metal ions [188,189]. This process removes heavy metals from the solution, preventing them from spreading further and posing a threat to aquatic life and human health. Many functionalized hydrogels incorporate stimulus-responsive mechanisms that trigger a measurable response upon binding heavy metals [190]. These responses can include:

(i) Colorimetric change: a change in color of the hydrogel or incorporated indicator molecules can signal the presence of heavy metals. This allows for easy visual detection, even in field tests [191].
(ii) Swelling response: the binding process can cause the hydrogel to swell or shrink. This change in volume can be measured and correlated with the concentration of the captured metal ions, providing a quantitative assessment of the contamination level [192].
(iii) Conductometric response: The presence of charged metal ions can alter the electrical conductivity of the hydrogel. This change can be measured electronically to quantify the amount of metal ions adsorbed [193].

**Figure 8:**
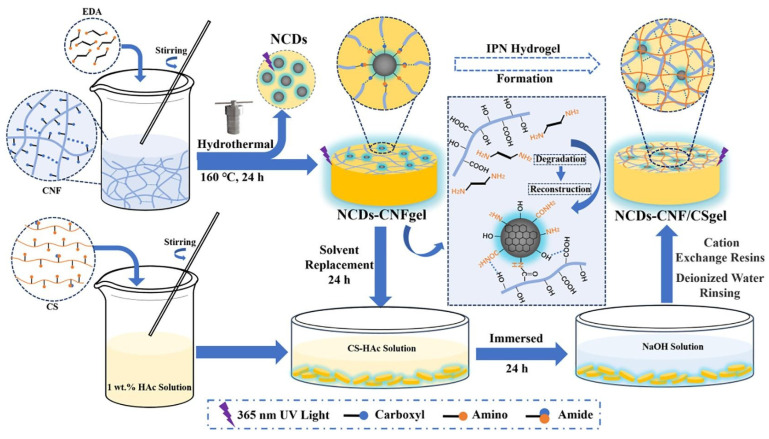
Schematization of the fluorescent NCDs-CNF/CSgel development. Reprinted with permission from Ref. [179]. Copyright 2022, Xueqi Chen, Chemical Engineering Journal, Elsevier.

To address the issue of heavy metal pollution in water sources, researchers have developed a novel, eco-friendly hydrogel material by combining self-assembled nitrogen-doped carbon dots (NCDs) with cellulose nanofibrils (CNF) and chitosan (CS) to create an interpenetrating polymer network (IPN). This hybrid material, known as NCDs-CNF/CSgel, was designed for simultaneous fluorescent detection and adsorption of Cu(II) cation and Cr(VI) anion. The NCDs were first cross-linked with the CNF network through a low-temperature hydrothermal process, followed by solvent replacement to incorporate the CS network, as depicted in Figure 8.

The findings revealed that NCDs-CNF/CSgel had a broad linear range of fluorescence response for Cu (II) (with a linear range of 50–1000 mg/L and a detection limit of 40.3398 mg/L) and better sensitivity and selectivity for Cr (VI) (with a linear range of 1–50 mg/L and a detection limit of 0.7093 mg/L). Additionally, the NCDs-CNF/CSgel revealed strong adsorption capabilities of 148.30 mg/g for Cu (II) and 294.46 mg/g for Cr (VI), respectively [179]. 

**Figure 9:**
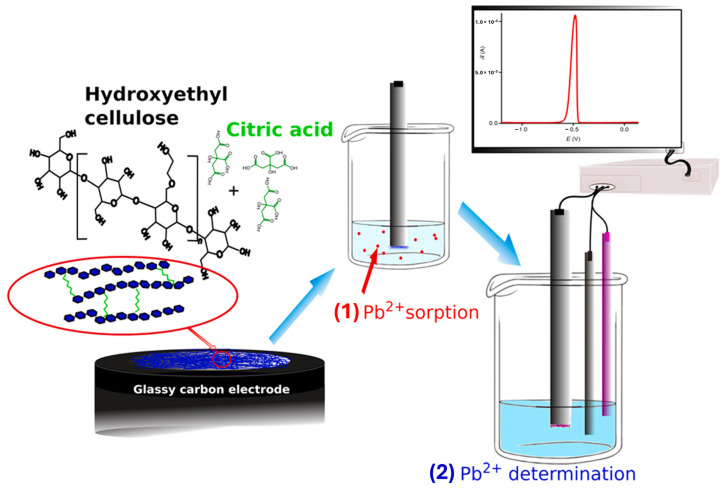
Representation of cellulose derivatives cross-linked by citric acid on the electrode surface as a heavy metal sorption (1)/sensing (2) matrix. Reprinted with permission from Ref. [180]. Copyright 2022, Jitka Sotolářová, Colloids and Surfaces A: Physicochemical and Engineering Aspects, Elsevier.

Modification of cellulose derivatives with citric acid increases sorption capacity towards metal ions, which has been widely used for heavy metal sequestration. In this regard, Sotolářová et al. performed the in-situ coating of a carbon electrode using citric acid-cross-linked cellulose derivatives. In particular, electrodes were modified by casting aqueous solutions of cellulose derivatives (hydroxyethyl cellulose, HEC, and thermally pre-treated carboxymethyl cellulose, (T-CMC)) mixed with citric acid (CA) on the electrode surface, followed by thermal treatment (110 °C, 1 h) to produce the desired HEC-CA and T-CMC-CA hydrogels coated on glassy carbon electrodes (Figure 9). 

The hydrogel-modified electrodes were applied for the determination of lead in solution and in real samples using the absorption of lead ions, followed by stripping voltammetry performed in a separate electrochemical cell. The detection limits of the sensors prepared from T-CMC-CA and HEC-CA were 1.13 and 0.4 mg·L^−1^, respectively, but they were improved to 0.75 and 0.15 mg·L^−1^ when the absorption step was prolonged from 1 to 5 min [180].

#### 3.2.2. Organic Pollutant Sensing

Organic pollutant hydrogel sensing has the potential to revolutionize water quality monitoring by providing a rapid, sensitive, and potentially field deployable approach. 

Research in this field is ongoing, with scientists exploring ways to improve the efficiency, stability, and reusability of hydrogels, as well as different methods for their preparation.

For example, solution casting, because of its non-toxicity and biocompatibility, was used by Katowah et al. to create chitosan/polyvinyl alcohol (CS/PVA) nanocomposite hydrogels. The optimum mixture was chosen and cross-linked with glutaraldehyde, followed by the addition of graphene oxide nanosheets (GO NS) and cerium-doped zinc oxide nanoparticles (Ce-ZnO NPs) to form nanocomposite hydrogel films (NCs). The synthesized composites were also evaluated for sensitivity towards methyl orange (MO) dye based on the quartz crystal microbalance (QCM) technique at different pH values of MO solution. QCM experiments demonstrated that the CS/PVA, CS/PVA/GO, and CS/PVA/GO/Ce-ZnO film-based sensors performed similarly at pH 7 and 11. The sensitivity of films to MO monitoring was ordered as CS/PVA/GO/Ce-ZnO > CS/PVA/GO > CS/PVA vs. contact time. These findings revealed that the addition of GO NS and Ce-ZnO NPs in the CS/PVA/GO/Ce-ZnO film-based QCM sensor boosted its high potential for MO dye sensing by providing the highest surface area and active site availability [181].

**Figure 10:**
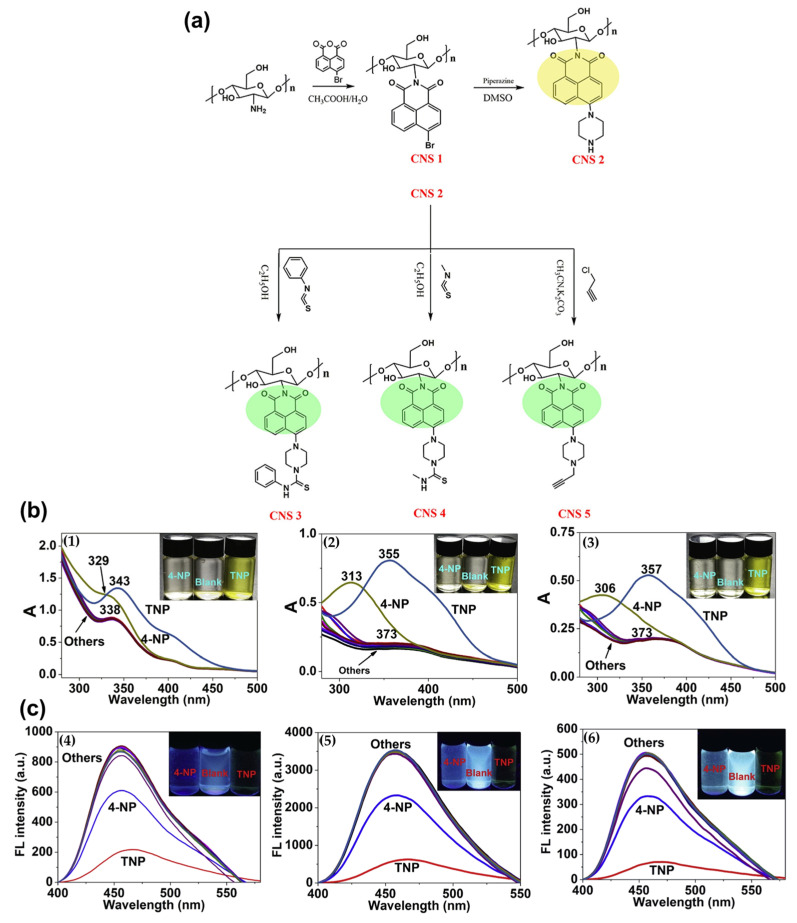
Synthesis of the three fluorescent chitosan polymers (**a**) and UV-vis absorption (**b**) of CNS 3 (1), CNS 4 (2), and CNS 5 (3) (0.25 μM) in 1% HAc/H_2_O after adding nitro compounds (2 sensor equivalents). Insert a snapshot of 4-NP and TNP with a blank sample under natural light. Fluorescence intensity changes (**c**) for CNS 3 (4), CNS 4 (5), and CNS 5 (6) (0.25 μM) in 1% HAc/H_2_O after adding nitro compounds (5 equivalents of the sensor); λex = 360 nm. Reprinted with permission from Ref. [182]. Copyright 2022, Jitka Sotolářová, Colloids and Surfaces A: Physicochemical and Engineering Aspects, Elsevier.

Among the many toxic organic dyes, nitroaromatic compounds like 2, 4, 6-trinitrophenol stand out for their significant biological toxicity and explosive potential, making it crucial to detect this specific compound. Xiong et al. have developed three fluorescent chitosan-based sensors that can detect 2, 4, 6-trinitrophenol (TNP) and/or p-nitrophenol (4-NP). The researchers used chitosan as the matrix and naphthalimide as the fluorophore, creating the following three successful sensors: CNS 3, CNS 4, and CNS 5 (Figure 10a).

When 2, 4, 6-trinitrophenol (TNP) and/or p-nitrophenol (4-NP) were present in the sample, the fluorescent chitosan sensors demonstrated significant fluorescence quenching (as shown in Figure 10b,c). Notably, CNS 4 exhibited the highest sensitivity among the three chitosan-based sensors, with a detection limit as low as 0.28 μM for TNP and 1.20 μM for 4-NP [182]. 

Besides these approaches, the development of new enzymes targeting a wider range of pollutants is an active area of research [194,195]. This technology can play a crucial role in safeguarding our water resources from harmful organic contaminants and ensuring clean water for all. Enzyme-immobilized hydrogels facilitate the detection of organic pollutants through enzymatic reactions, offering rapid and sensitive detection of organic pollutants in water. Enzymes are inherently highly specific catalysts. 

The enzymes, chosen to break down or react with specific organic pollutants like pesticides, phenols, or hydrocarbons, are not simply dumped into the water [196,197], but they are instead immobilized within the three-dimensional network of the hydrogel. This hydrogel acts as a scaffold, holding the enzymes in place and protecting them from harsh environmental conditions. When the contaminated water comes into contact with the hydrogel, the target pollutants encounter the immobilized enzymes [198]. These enzymes then catalyze specific reactions, breaking down or transforming the pollutants [199]. 

There are two main ways to detect the presence of pollutants based on the enzymatic reactions: in some cases, the hydrogel incorporates indicator dyes that change color upon the breakdown of the pollutant by the enzyme, and this color change provides a visual signal for the presence of the contaminant; in other designs, the enzymatic reaction might generate a specific product [200,201]. By measuring the concentration of this product, scientists can quantify the amount of pollutant present in the water sample. This offers a more quantitative approach to detection.

As an example, acetylcholinesterase was immobilized by Kestwal et al. in a new fenugreek hydrogel-agarose matrix containing gold nanoparticles for the detection of carbamate pesticides. 

Pesticides are commonly employed in agriculture, and their aqueous solubility determines whether they remain in the soil or enter surface and groundwater. Compounds arising from pesticide breakdown can persist in animals, crops, and water sources, becoming more concentrated as they ascend the food chain. Transparent thin films with high mechanical strength and stability were created using 2% fenugreek hydrogel and 2% agarose. Acetylcholinesterase immobilization on the membrane resulted in a high enzyme retention efficiency (92%) and a much longer enzyme shelf life (a half-life of 55 days). 

This immobilized enzyme-gold nanoparticle photometric dip-strip system identified carbamates such as carbofuran, oxamyl, methomyl, and carbaryl at limits of detection of 2, 21, 113, and 236 nM (S/N = 3), respectively. Furthermore, the biosensor demonstrated good testing capabilities when utilized to identify carbamates in a variety of fruit and vegetable samples [184].

Pharmaceutical substances have the aim of causing a biological response in certain species. They may, however, generate a biological response in non-specific species when exposed to ambient levels, causing possible human health risks and environmental repercussions. In this regard, the effective and real-time detection and removal of such substances is increasingly studied by researchers [202]. As already mentioned, rare earth elements have a high potential for improving the removal of hazardous organic substances from aqueous solutions and also in wastewater [203].

With this in mind, Aggarwal et al. developed a dual-responsive guava leaf (Psidium guajava) and Cerium-MOF biocomposite for tetracycline antibiotic adsorption and fluorescence detection. The Ce-MOF nanoparticles were created utilizing a hydrothermal method using benzene-1,4-dicarboxylic acid as the organic ligand. Guava leaf powder (0.1, 0.5, and 1.0 g) was added to the Ce-MOF synthetic procedure to produce the required composite material. The finished product was identified as Ce-MOF/x-GLP, where x represents the amount (in g) of GLP added. The experimental results revealed a high tetracycline antibiotic adsorption capacity of 81.30, 75.18, and 63.69 mg/g for doxycycline (DC), minocycline (MC), and tetracycline (TC), respectively, by the Ce-MOF/0.5-GLP composite. In addition, the manufactured bio-based Ce-MOF/0.5-GLP composite was used for selective fluorescence detection of such compounds, demonstrating high selectivity and sensitivity with low detection limit values of 94.3 nM, 71.4 nM, and 74.2 nM for DC, MC, and TC [183].

#### 3.2.3. Nutrient Monitoring

Nutrients like nitrates and phosphates are essential for aquatic life, but their excessive levels can lead to harmful algal blooms and disrupt ecosystems [204]. Here is where hydrogels come into play as a promising tool for nutrient monitoring in water [205]. Hydrogels can be designed to respond specifically to changes in nutrient levels or pH, providing valuable data for effective water management [206], as in the following three main classes:

(i) pH-responsive hydrogels can incorporate polymers that change their properties depending on the surrounding pH [207]. Since most natural water bodies have a relatively neutral pH, a shift towards acidity or alkalinity can indicate changes in nutrient levels. For example, an increased nitrate concentration can lead to a decrease in pH. The hydrogel might swell, shrink, or exhibit a change in electrical conductivity, depending on the chosen design [208,209]. These changes can be measured and correlated with the overall pH of the water, indirectly indicating potential nutrient imbalances.
(ii) Ion-selective hydrogels can contain specific molecules called ionophores that can selectively bind with targeted nutrient ions, like phosphates or nitrates. When the target ions come into contact with the hydrogel, the ionophores bind them [210]. The binding process can alter the hydrogel electrical properties, such as conductivity [211]. This change can be measured electronically and directly quantified to determine the concentration of the target nutrient ion.
(iii) Colorimetric hydrogels can include chromophores for the detection of nutrients by color variations [212].

**Figure 11:**
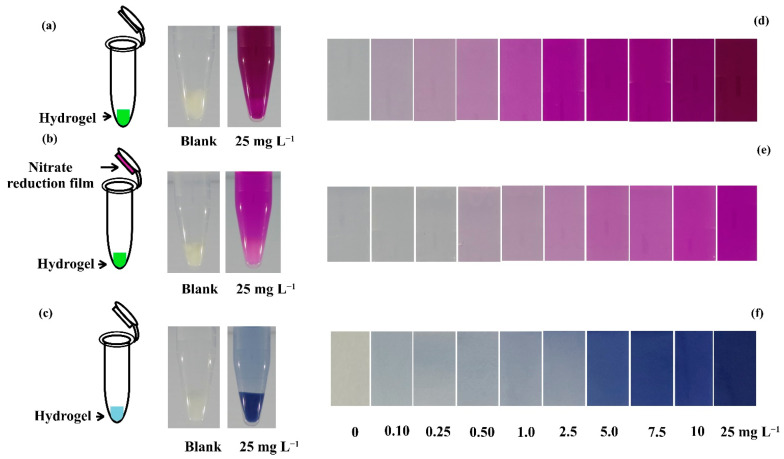
Schematization of in-tube hydrogel test kits for detecting (**a**) nitrite, (**b**) nitrate, and (**c**) phosphate, as well as colorimetric products derived by testing standard solutions with the (**d**) nitrite, (**e**) nitrate, and (**f**) phosphate test kits. Reprinted from Ref. [185]. Copyright 2022, Worawit Wongniramaikul, Molecules, MDPI.

A portable colorimetric hydrogel test kit was developed by Wongniramaikul et al. to detect nitrite, nitrate, and phosphate in water on-site (Figure 11).

In particular, a water-based hydrogel with Griess reagent was created at the bottom of a 1.5 mL plastic tube (Figure 11a) to detect nitrites. A nitrate reduction film made of zinc powder trapped in tapioca starch was placed on the inner lid of a second 1.5 mL plastic tube to be used in combination with the Griess-doped hydrogel (Figure 11b). Meanwhile, a molybdenum blue-based reagent was embedded in a poly(vinyl alcohol) hydrogel matrix at the bottom of a third 1.5 mL plastic tube to detect phosphates (Figure 11c).

These test kits were evaluated for their ability to detect nitrite, nitrate, and phosphate in water, and they showed excellent performance when combined with mobile digital image colorimetry (DIC) for on-site nutrition assessment. 

The kits are also environmentally friendly because the polymers used, PVA and tapioca starch, are biodegradable. The detection limits for nitrite, nitrate, and phosphate were 0.02, 0.04, and 0.14 milligrams per liter, respectively, with high accuracy (less than 4.8%) and precision (less than 1.85% relative standard deviation) [185].

As a result, the use of hydrogel-based nutrient monitoring provides a sustainable and cost-effective way to ensure the health of aquatic ecosystems. By continuously providing sensitive data, these intelligent materials enable us to make informed decisions, ultimately contributing to a cleaner and healthier water environment in the future. 

#### 3.2.4. pH and Chemical Monitoring

pH-responsive hydrogels serve as pH sensors for monitoring acid–base variations in wastewater, while chemically responsive hydrogels detect specific chemical species or pollutants through reversible interactions, expanding the sensor capabilities for comprehensive water quality assessment [213]. pH-responsive hydrogels act as sentinels, continuously monitoring acid–base variations within the wastewater. These hydrogels incorporate polymers that change their properties based on the surrounding pH [214]. As the pH fluctuates, the hydrogel might expand or contract, causing a measurable physical change or changing its electrical conductivity due to the presence of charged groups within the polymer that ionize at different pH levels [215]. Some hydrogels can also include indicator dyes that change color based on the surrounding pH, providing a visual cue for real-time monitoring [216,217]. While pH-responsive hydrogels are crucial, wastewater often contains a complex mixture of pollutants. In this regard, chemically responsive hydrogels offer an extra layer of detection by targeting specific chemical species. These hydrogels have cavities with specific shapes and functionalities designed to match the target pollutants. The binding event can cause a measurable change in the hydrogel, such as swelling or a colorimetric response [218]. 

**Figure 12:**
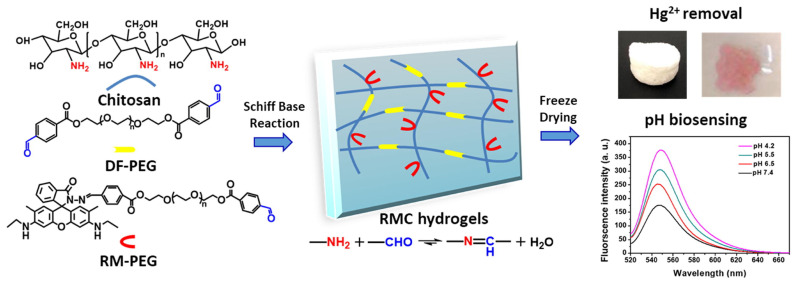
Illustration of the rhodamine-modified chitosan (RMC) hydrogels and their possible uses for colorimetric heavy metal detection and pH biosensing. Reprinted with permission from Ref. [186]. Copyright 2021, Xiaoyong Qiu, Journal of Colloid and Interface Science, Elsevier.

For these purposes, Qui et al. developed a multifunctional rhodamine-modified chitosan (RMC) hydrogel for Hg^2+^ adsorption with fluorescent turn-on properties by grafting a rhodamine-modified poly(ethylene glycol) benzaldehyde (RM-PEG) onto the hydrogel network, which serves as the fluorescence/colorimetric sensing receptor (Figure 12). RMC hydrogel can remove more than 96.5% of Hg^2+^ from an aqueous solution while exhibiting strong fluorescence and colorimetric changes. 

The hard and soft acid/base (HSAB) theory explains the excellent adsorption selectivity and colorimetric sensing method for Hg^2+^ in RMC hydrogel. The O atom in the hydroxyl and carbonyl groups, as well as the N atom in the amine/imine groups of RMC hydrogel, play an important role in Hg^2+^ adsorption, whereas the colorimetric response and fluorescence enhancement of the hydrogel after adsorption are attributed to the rhodamine moieties-specific spiro-lactam structure. Furthermore, significant fluorescence emission enhancement at 547 nm was found when pH values decreased, demonstrating that the RMC hydrogel fluorescent behavior is also H^+^ dependent. RM-PEG exhibited low cytotoxicity towards mouse embryonic fibroblast cells, and RMC hydrogel may be employed as a fluorescent pH indicator ranging from 4.2 to 7.4, suggesting RMC hydrogel has potential uses in biological diagnostics [186].

Therefore, by combining pH and chemical-responsive hydrogels, a more comprehensive picture of wastewater quality can be obtained. Integration with advanced spectroscopy or electrochemical methods could provide even more detailed information about the types and concentrations of pollutants present. This allows for better management of treatment processes and ensures efficient removal of a wider range of pollutants, paving the way for cleaner water, improved environmental protection, and sustainable water management practices.

## 4. Final Remarks and Future Perspectives

This review assessed various methods and techniques for addressing the challenges associated with using bio-based polymeric hydrogels in water treatment and sensing technologies. The reported studies specifically explored the use of diverse nanoparticles and cross-linking agents to enhance the mechanical properties and specificity/sensitivity of these bio-based hydrogels for detecting specific pollutants. The development of cost-effective production methods was also explored, including novel synthesis techniques that utilize secondary raw materials to reduce production costs and enhance scalability. Additionally, hydrogels with improved durability under environmental conditions and biodegradation rates that meet specific application demands were described. Table 4 provides a summary of the main benefits and drawbacks of several bio-based polymeric hydrogels employed for water treatment and sensing purposes.

As a matter of fact, the future of wastewater treatment looks increasingly promising, thanks to the design and development of innovative bio-based and stimuli-responsive hydrogels. As research advances, we can expect the creation of even more sophisticated sorbent-functional hydrogel-based materials and sensors with enhanced capabilities for removal, detection, and functionality. This review highlights scientists efforts to design novel bio-based hydrogel materials with improved sensitivity, selectivity, and regenerative capabilities. Moreover, integrating data transmission and machine learning may enable real-time monitoring and automated control of wastewater treatment plants, leading to a more sustainable and efficient approach to protecting water resources and remediating wastewater. Despite the progress made, there are still challenges to overcome, such as achieving precise control over responsiveness, improving mechanical strength and stability, and enhancing reversibility and biocompatibility. Future directions will include the development of multifunctional hydrogels responsive to multiple stimuli and the integration of advanced technologies like nanotechnology and 3D printing to translate these materials into efficient wastewater treatment applications. The potential of stimuli-responsive hydrogels is vast, opening opportunities for designing intelligent biomaterials with tailored properties and functions, which can lead to breakthroughs in wastewater management, drug delivery, tissue engineering, biosensing, and smart devices. Keeping research and innovation in this field will drive further progress and enable transformative applications in various areas, particularly in wastewater treatment and monitoring, leading to cleaner water, improved public health, and a more sustainable future.

## Figures and Tables

**Table 1:** Most recent composite/hybrid and bio-based hydrogels based on functional nano- and micro-dopant agents (i.e., nanoparticles, clays, and lignocellulosic derivatives) and comparison of their sorption performances towards different pollutants in water.

Bio-Polymeric Blend Composition	Nano-Micro-Dopant Agent	Pollutant Treated	Sorption Performances	Ref.
Karaya gum cross-link poly(acrylamide-co-acrylonitrile)	AgNPs	Crystal violet	Initial concentration: 50 mg/L; Adsorption capacity: 1000 mg·g^−1^ (pH = 8 and temperature 298 K)	[39]
Guar gum	Ag NPs@ MIL-100(Fe)	Methylene blue, silicone oil, cyclohexane, and canola oil	Initial MB concentration: 40 mg/L; removal: 100% Oil/water separation ability: 99.10% for silicone oil, 97.82% for cyclohexane, and 98.61% for canola oil	[40]
Chitosan hydrogel beads	La- and Ce-layered double hydroxide	Phosphate	Initial concentration: 200 mg P/L (pH 5); adsorption capacity: LaCa-LDH/CS and CeCa-LDH/CS of 149.5 and 174.6 mg P/g, respectively	[41]
Chitosan/partially hydrolyzed polyacrylamide	Graphene oxide nanosheets	Methylene blue	Initial concentration: 100 mg/L; adsorption capacity: 476.19 mg·g^−1^	[42]
Chitosan/carboxymethyl cellulose	Carbon dots	Heavy metal ions, detergents, and organic compounds	Contaminants: Cu^2+^, Ni^2+^, Ag^+^, and Cd^2+^; initial concentration: 0.01 M; removal: >99%	[43]
Gelatin	Bentonite	Pb(II)	Adsorption capacity: 47.16 mg/g (pH = 5)	[44]
Alginate/soybean extract	Hemp hurd and halloysite nanotubes	Methylene blue	Adsorption capacity: 49 mg/g	[45]
κ-carrageenan	Kaolinite and Fe_3_O_4_/Al_2_O_3_ core–shell nanoparticles	Congo red and Alizarin Red S	Adsorption capacity: 26.9 and 33.5 mg/g for Congo red and Alizarin Red S, respectively	[46]
Chitosan	Dodecylamine, dellite LVF, and bentonite	Paraquat	Adsorption capacity: 0.98, 0.94, and 0.99 mg/g for dodecylamine, dellite LVF, and bentonite modified chitosan beads	[47]
Alginate	Cellulose nanocrystals and Fe^2+^	Tetracycline	Adsorption capacity: 741.66 mg·g^−1^	[48]
Starch/itaconic acid/acrylic acid	Cellulose nanofibers and zinc oxide nanoparticles	Cu(II) and Fe(II) ions	Adsorption capacity: 122 and 70 mg/g for Cu(II) and Fe(II) ions, respectively	[49]
Acrylamide-based	Nanoscale Zero-Valent Iron (nZVI)	Cr(VI)	Adsorption capacity: 310.86 mg·g^−1^	[50]
Cellulose and guar gum	*Typha angustifolia* biochar	Cu^2+^, Co^2+^, and methylene blue	Adsorption capacity: 805.45, 772.52, and 598.28 mg·g^−1^ for Cu^2+^, Co^2+^, and methylene blue	[51]
Carboxymethyl cellulose grafted acrylamide	Porous carbon (from bagasse) and citric acid-modified magnetite	Pb(II) and methylene blue	Adsorption capacity: 294.1 and 222.2 mg/g for Pb-ions and methylene blue, respectively	[52]

**Table 2:** Stimulus, mechanism, and related examples of common types of stimuli-responsive hydrogels.

Stimulus	Hydrogel Type	Applications	Mechanism	Example	Ref.
Temperature	Thermo-responsive	Medicine Biotechnology	Changes in swelling behavior or sol–gel transition in response to temperature variations	PNIPAM hydrogel PNIPAM-PEG	[138,139,140,141,142]
pH	Acid or basic	Medicine Agriculture Environmental monitoring	Changes in swelling behavior or structural conformation in response to changes in pH	PDMAEMA/ crystalline nanocellulose (CNC)	[151]
Light	Photo-Responsive	Medicine Biotechnology Advanced materials	When exposed to particular light wavelengths, photo-responsive moieties can undergo reversible photoisomerization or photolysis, which can alter the structure and mechanical strength of hydrogels	Polysaccharide hybrid hydrogels (arylazopyrazole-modified hyaluronic acid (HA-AAP), guanidinium functionalized β-cyclodextrin (Guano-CD), and laponite clay (LP) Agarose hydrogel matrix containing gold nanoparticles (AuNPs) covered with polymer poly(methacryloxyethyl trimethyl ammonium chloride [P(METAC)]	[158,160,161]
Electrochemical	Redox-Responsive	Drug delivery Regenerative medicine Biodetection	Redox-responsive hydrogels respond to changes in the oxidation state of their environment	Carboxymethyl cellulose nanocrystals and L-cysteine-based hydrogel	[163]
Target molecules	Molecular Recognition-Responsive	Medicine Biotechnology Environmental monitoring Advanced materials	When particular molecules are present, molecular recognition-responsive hydrogels react, providing very focused and selective reactions	Aptamers-functionalized Fe_3_O_4_–Ag Janus nanoparticles into cellulose hydrogel	[174]

**Table 3:** Most recent composite/hybrid and bio-/water-based stimuli-responsive hydrogels for water contaminants sensing and monitoring.

Polymeric Blend Composition	Nano-Micro-Dopant Agent/Cross-Linker	Pollutant Detected by Revealing Approach	Results	Ref.
Chitosan	Nitrogen-doped carbon dots and cellulose nanofibrils	Cu(II) and Cr(VI) by fluorescence	Linear range: 50–1000 mg/L and 1–50 mg/L for Cu (II) and Cr (VI), respectively Detection limit: 40.3398 mg/L and 0.7093 mg/L for Cu (II) and Cr (VI), respectively	[179]
Hydroxyethyl cellulose (HEC) and thermally pre-treated carboxymethyl cellulose (T-CMC)	Citric acid	Pb(II) by electrochemical method	Detection limit: 0.39 mg·L^−1^ Sensitivity: 9.91 μA·L·mg^−1^	[180]
Chitosan/polyvinyl alcohol	Graphene oxide nanosheets and cerium-doped zinc oxide nanoparticles	Methyl orange by quartz crystal microbalance	Sensitivity: 25 ng Response time: 10 min	[181]
Chitosan	Naphthalimide derivatives	2, 4, 6-trinitrophenol (TNP), and/or p-nitrophenol (4-NP) by fluorescence	Limit of detection: 0.28 μM for TNP and 1.20 μM for 4-NP	[182]
Guava leaf (Psidium guajava)	Cerium-MOF	Doxycycline (DC), minocycline (MC), and tetracycline (TC)	Sorption capacities: 81.30, 75.18, and 63.69 mg/g for DC, MC, and TC, respectively Limit of detection: 94.3 nM, 71.4 nM, and 74.2 nM for DC, MC, and TC, respectively	[183]
Fenugreek hydrogel-agarose	Acetylcholinesterase and gold nanoparticles	Carbofuran, oxamyl, methomyl, or carbaryl by the photometric dip-strip system	Limit of detection: 2, 21, 113, and 236 nM for carbofuran, oxamyl, methomyl and carbaryl, respectively	[184]
Poly(vinyl alcohol)	Griess reagent, molybdenum blue-based reagent, and zinc powder entrapped within a tapioca starch film	Nitrite, nitrate, and phosphate by on-mobile digital image colorimetry	Limit of detection: 0.02, 0.04, and 0.14 mg·L^−1^ for nitrite, nitrate, and phosphate, respectively	[185]
Chitosan	Rhodamine-modified poly (ethylene glycol) benzaldehyde	Hg(II) and pH by colorimetry	Hg(II) removal rate: 96.5%; Hg(II) initial concentration: 158 mg·L^−1^ pH variations: from 4.2 to 7.4	[186]

**Table 4:** List of the advantages and limitations of the use of bio-based polymeric hydrogels in water remediation and sensing applications.

Advantages	Limitations
Sustainability and environmental friendliness Bio-based hydrogels can be derived from renewable biomaterials, such as polysaccharides (e.g., cellulose and chitosan) or proteins (e.g., collage and, gelatin), contributing to efforts towards sustainability and circular economy principles [189]	Weak mechanical properties Many bio-based hydrogels have low mechanical strength, which can limit their durability and practical application in dynamic water environments; moreover, continuous swelling and shrinking with changes in environmental conditions (e.g., pH and temperature) can affect their structural integrity and functionality [219]
Biocompatibility Hydrogels derived from renewable biomaterials are inherently biocompatible, minimizing adverse effects when deployed in aquatic environments or biological treatment systems and not inducing adverse reactions when in contact with biological tissues or fluids [200]	Production costs Bio-based hydrogels can be more expensive to produce than synthetic hydrogels, limiting their economic viability [220]
Biodegradability Bio-based hydrogels can be broken down into simpler molecules by biological processes over time, reducing the risk of long-term accumulation and minimizing environmental pollution [221]	Chemical stability Biodegradation of bio-based hydrogels exposed to natural environmental conditions may reduce their effectiveness and lifespan [19]
Stimuli-responsive and smart properties Stimuli-responsive hydrogels undergo reversible changes in their structure, properties, or behavior in response to specific external stimuli, such as changes in temperature, pH, light, or the presence of certain ions or molecules. The high water content of hydrogels enhances analyte diffusion and facilitates sensitive detection of trace contaminants in wastewater samples [208,211,222]	Biofouling Biological fouling that can occur on the hydrogel surface can reduce their sorption/detection performances, particularly in natural water bodies [223,224]
Adaptability and multifunctionality Hydrogel properties can be tailored to meet specific requirements, including mechanical strength, responsiveness, contaminant sorption, and compatibility with sensor transduction mechanisms [225,226,227]	
Selectivity Functionalization of bio-based polymeric hydrogels allows for specific sorption/detection of target analytes, such as heavy metals, organic pollutants, and pathogens, amidst complex wastewater matrices [33,228,229]	

## Data Availability

Not applicable.
